# Supplementary figures and images for: mFABIO: An integrative multi-tissue TWAS fine-mapping approach to prioritize potentially causal genes and tissues underlying binary traits
Source: PLoS Genet. 2026 May 27;22(5):e1012157. doi: 10.1371/journal.pgen.1012157 (PMC13225670; doi:10.1371/journal.pgen.1012157)

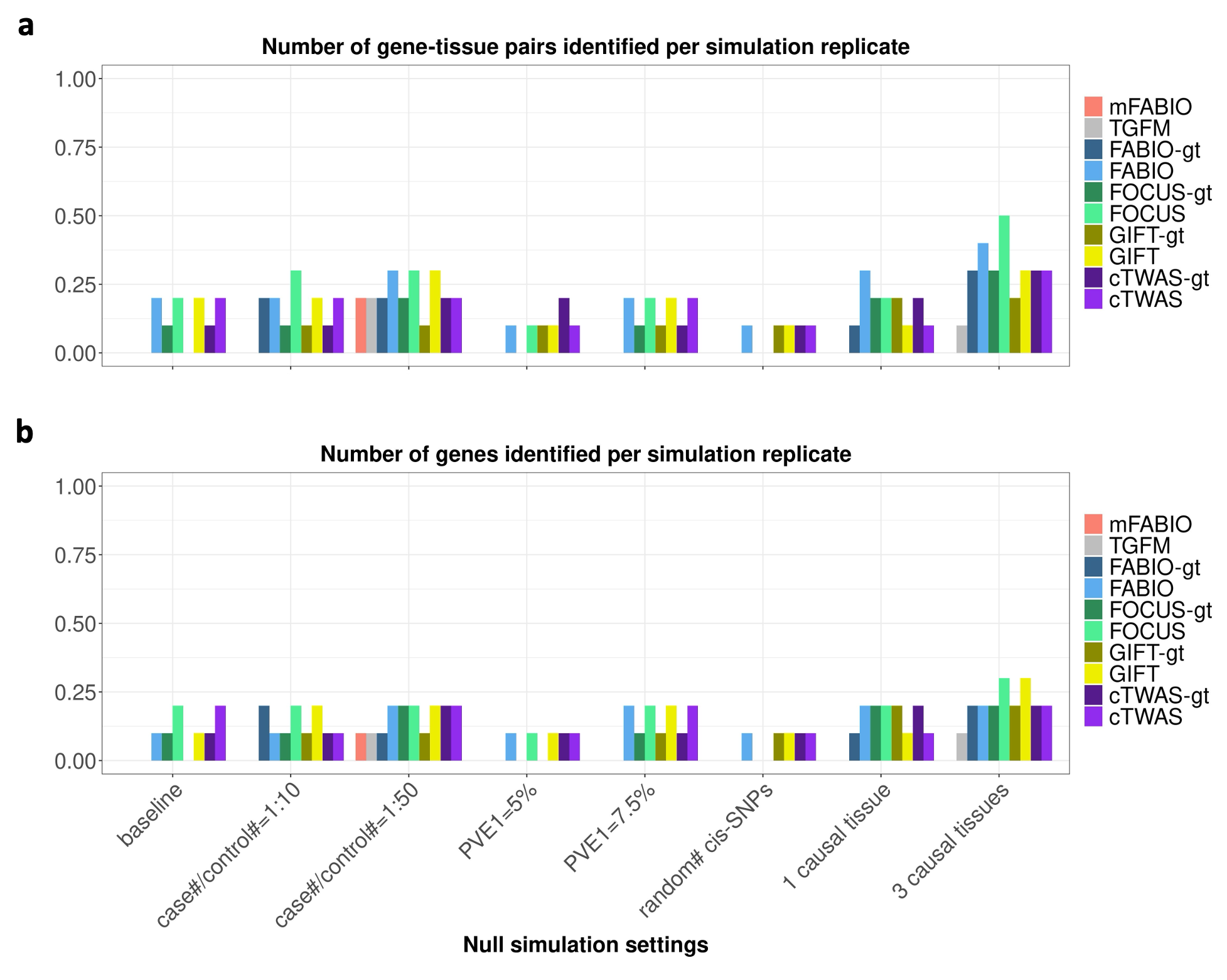

Supplement: S1 Fig — (a) The average number of false positive gene-tissue pairs per simulation replicate across null settings using an estimated FDR threshold of 0.05. (b) The average number of false positive genes per simulation replicate across null settings using an estimated FDR threshold of 0.05. (TIFF) [file pgen.1012157.s001.tiff]

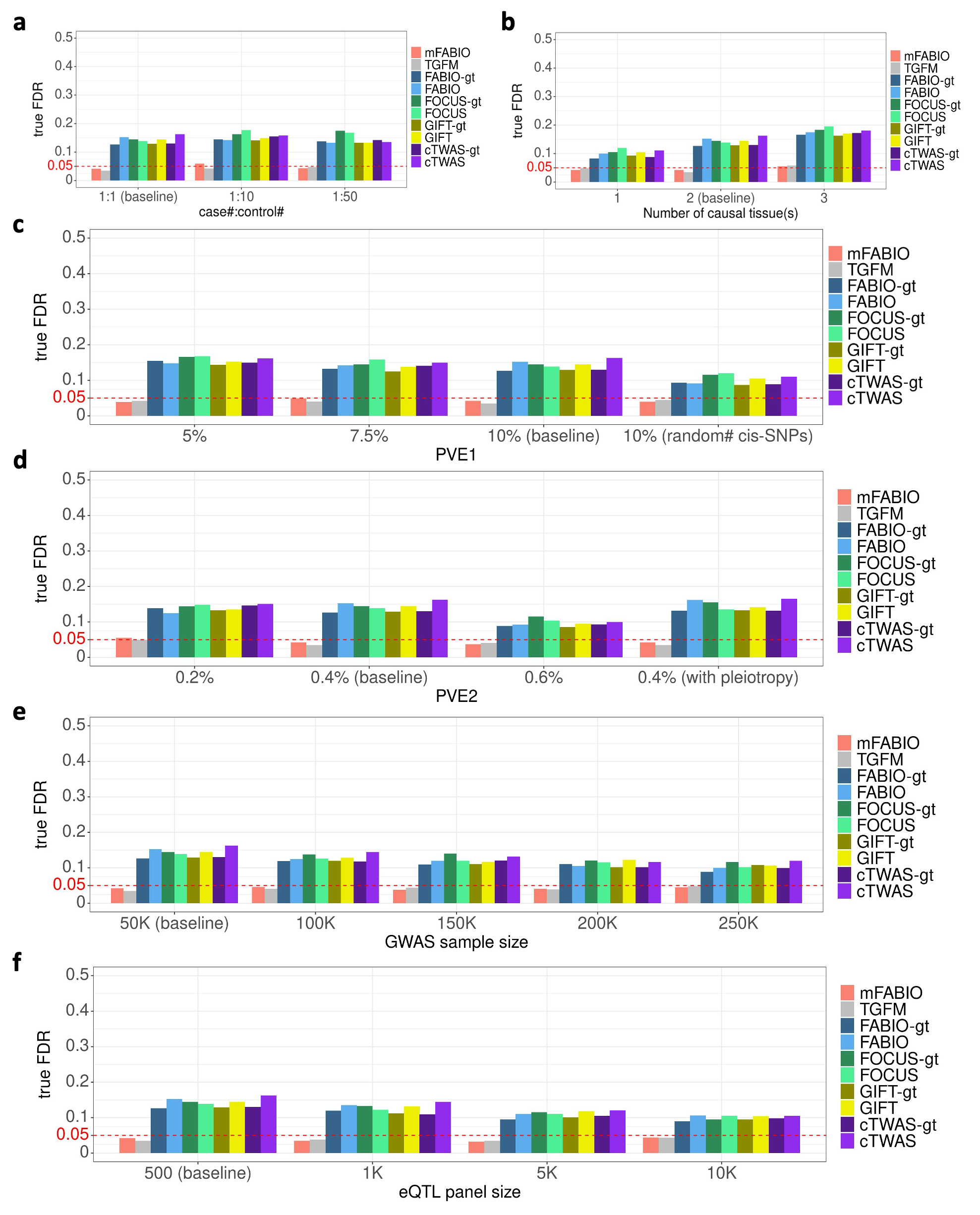

Supplement: S2 Fig — We calculated the true FDR under an estimated FDR threshold of 0.05 to evaluate the calibration of the methods in different simulation settings: (a) different case:control ratios; (b) different numbers of causal tissue(s); (c) different proportions of gene expression variance explained by genetic effects (PVE1) and different numbers of causal cis-SNPs; (d) different proportions of the phenotype’s variance explained by causal gene-tissue pairs (PVE2); (e) different GWAS sample sizes; (f) different eQTL sample sizes. (TIFF) [file pgen.1012157.s002.tiff]

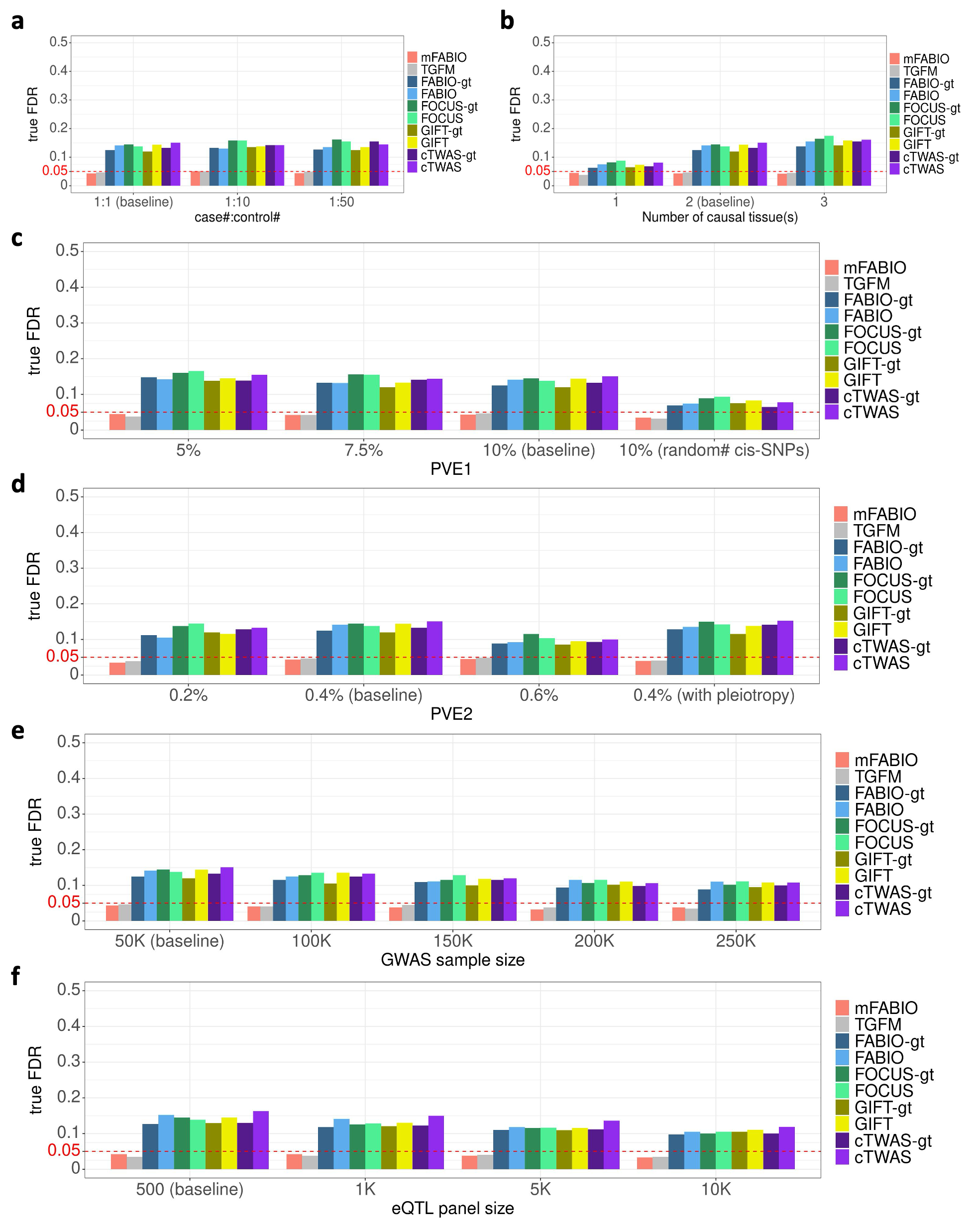

Supplement: S3 Fig — We calculated the true FDR under an estimated FDR threshold of 0.05 to evaluate the calibration of the methods in different simulation settings: (a) different case:control ratios; (b) different numbers of causal tissue(s); (c) different proportions of gene expression variance explained by genetic effects (PVE1) and different numbers of causal cis-SNPs; (d) different proportions of the phenotype’s variance explained by causal gene-tissue pairs (PVE2); (e) different GWAS sample sizes; (f) different eQTL sample sizes. (TIFF) [file pgen.1012157.s003.tiff]

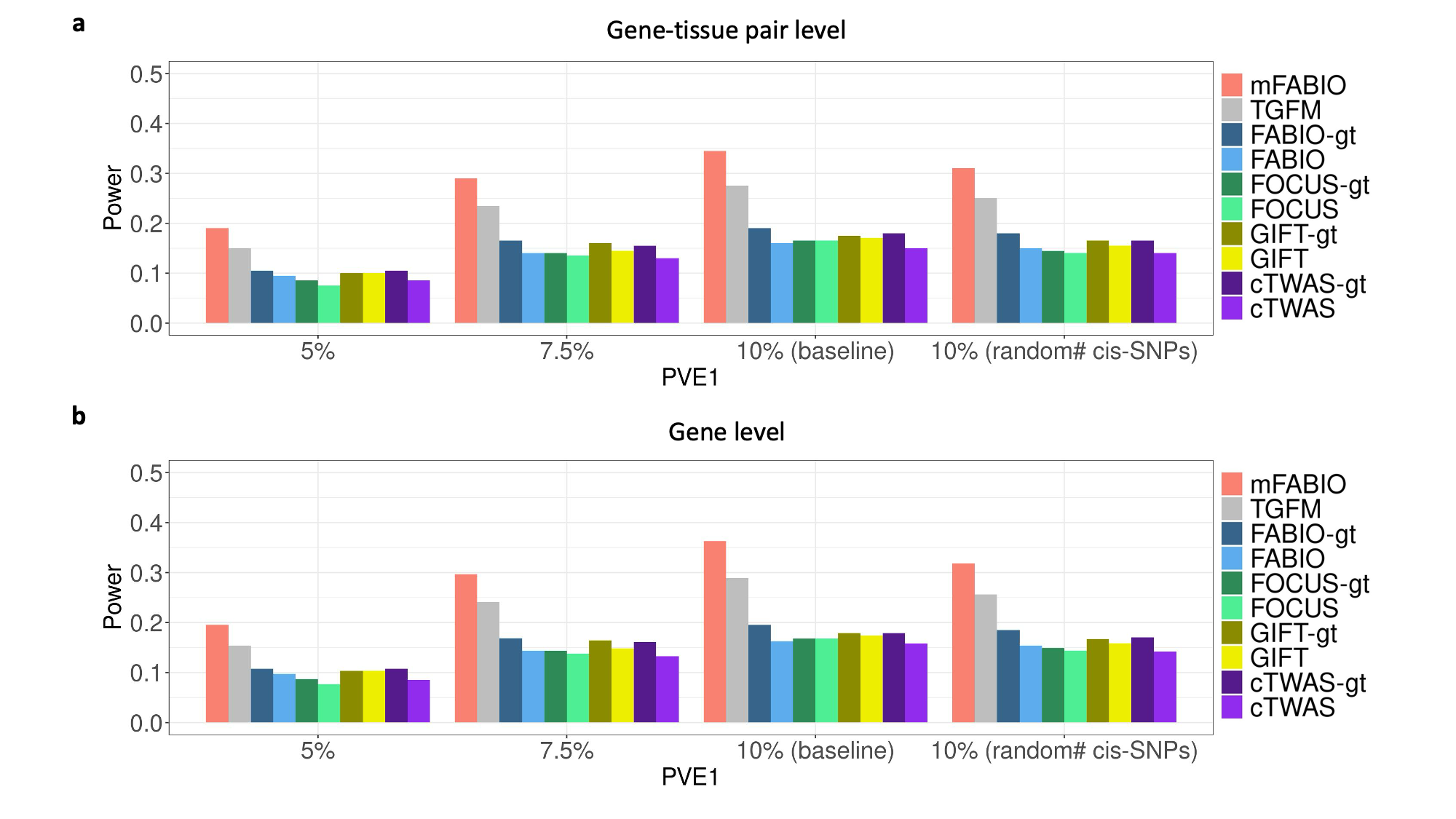

Supplement: S4 Fig — (a) Results at gene-tissue pair level. (b) Results at gene level. (TIFF) [file pgen.1012157.s004.tiff]

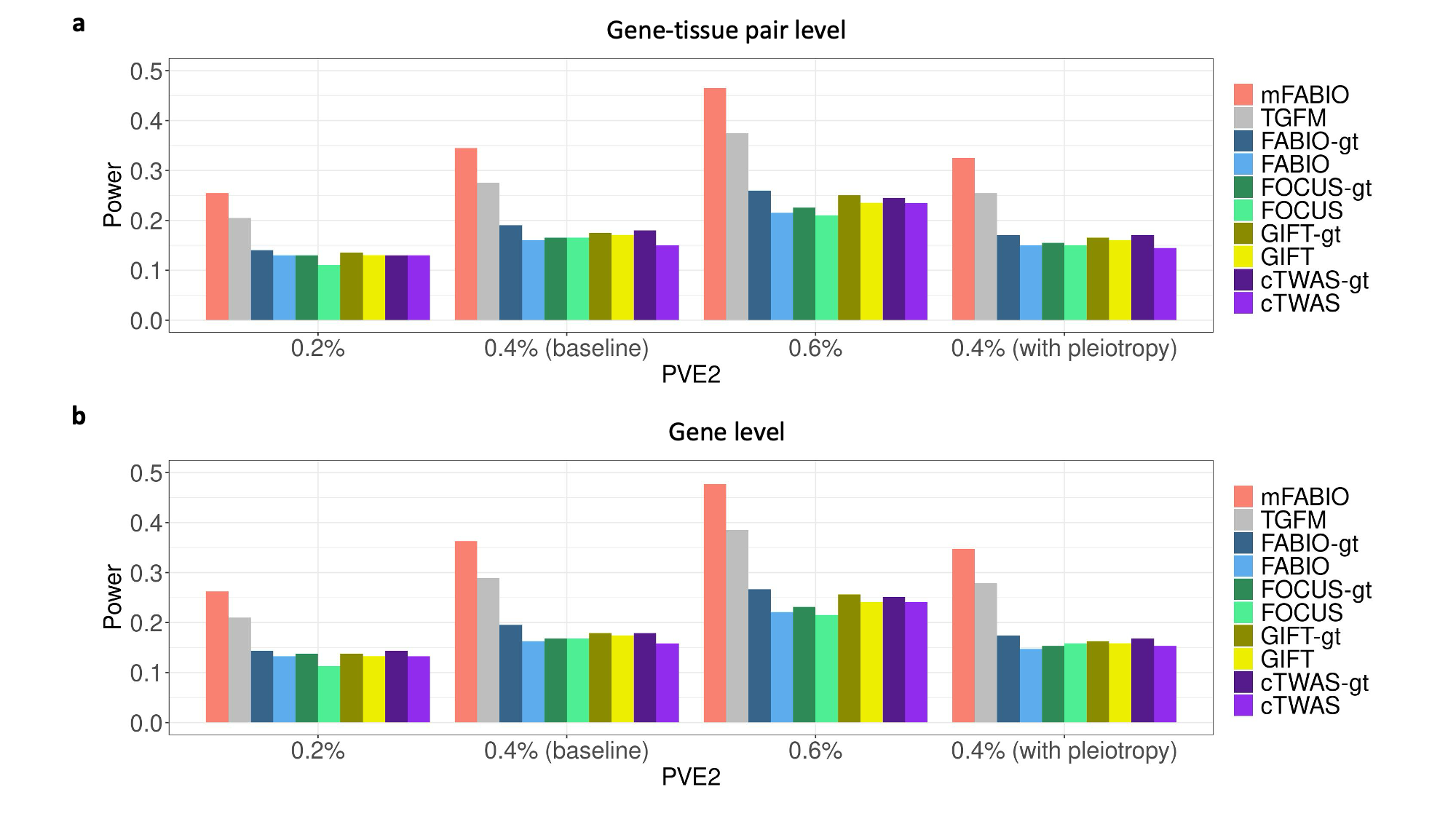

Supplement: S5 Fig — (a) Results at gene-tissue pair level. (b) Results at gene level. (TIFF) [file pgen.1012157.s005.tiff]

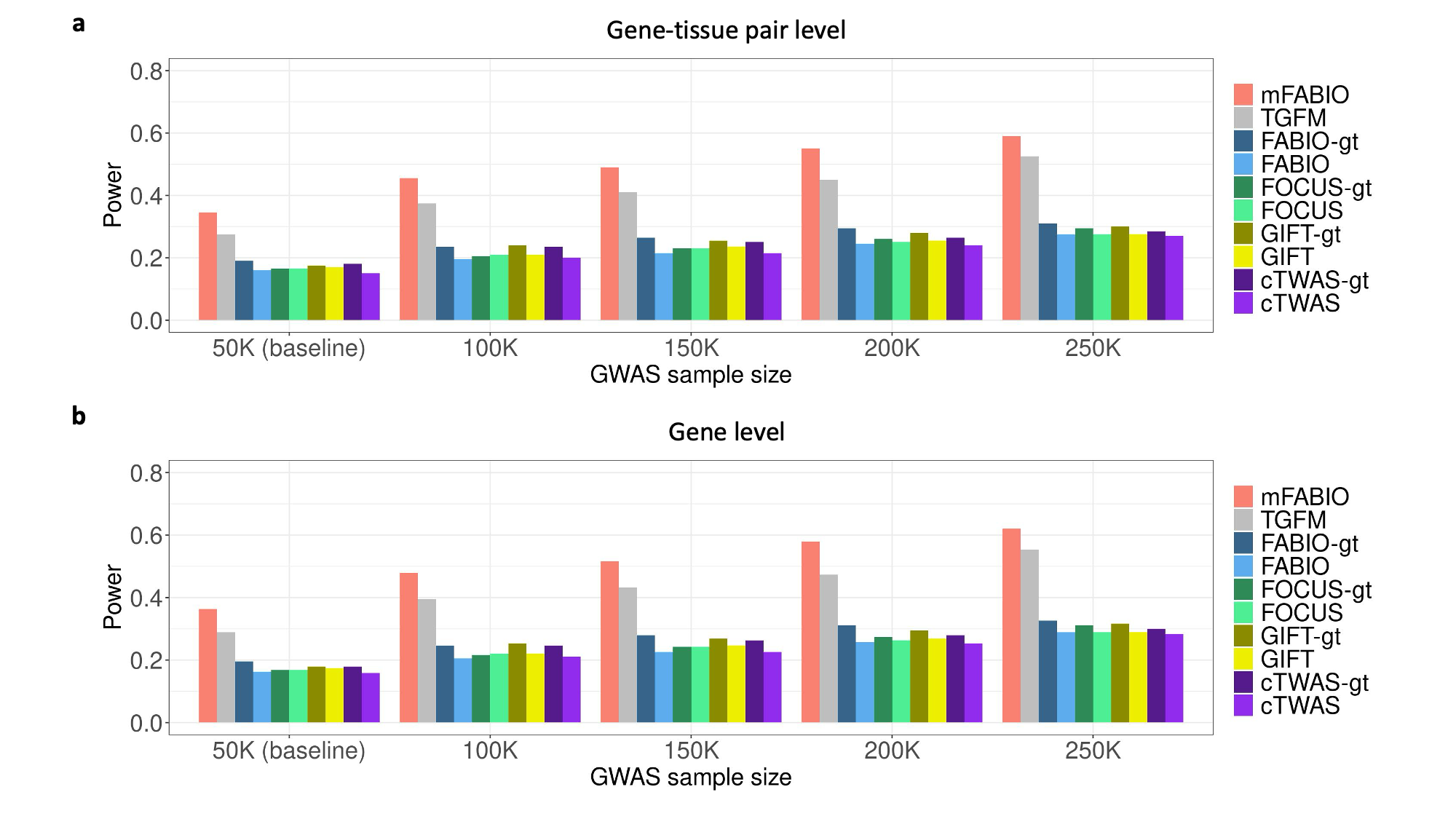

Supplement: S6 Fig — (a) Results at gene-tissue pair level. (b) Results at gene level. (TIFF) [file pgen.1012157.s006.tiff]

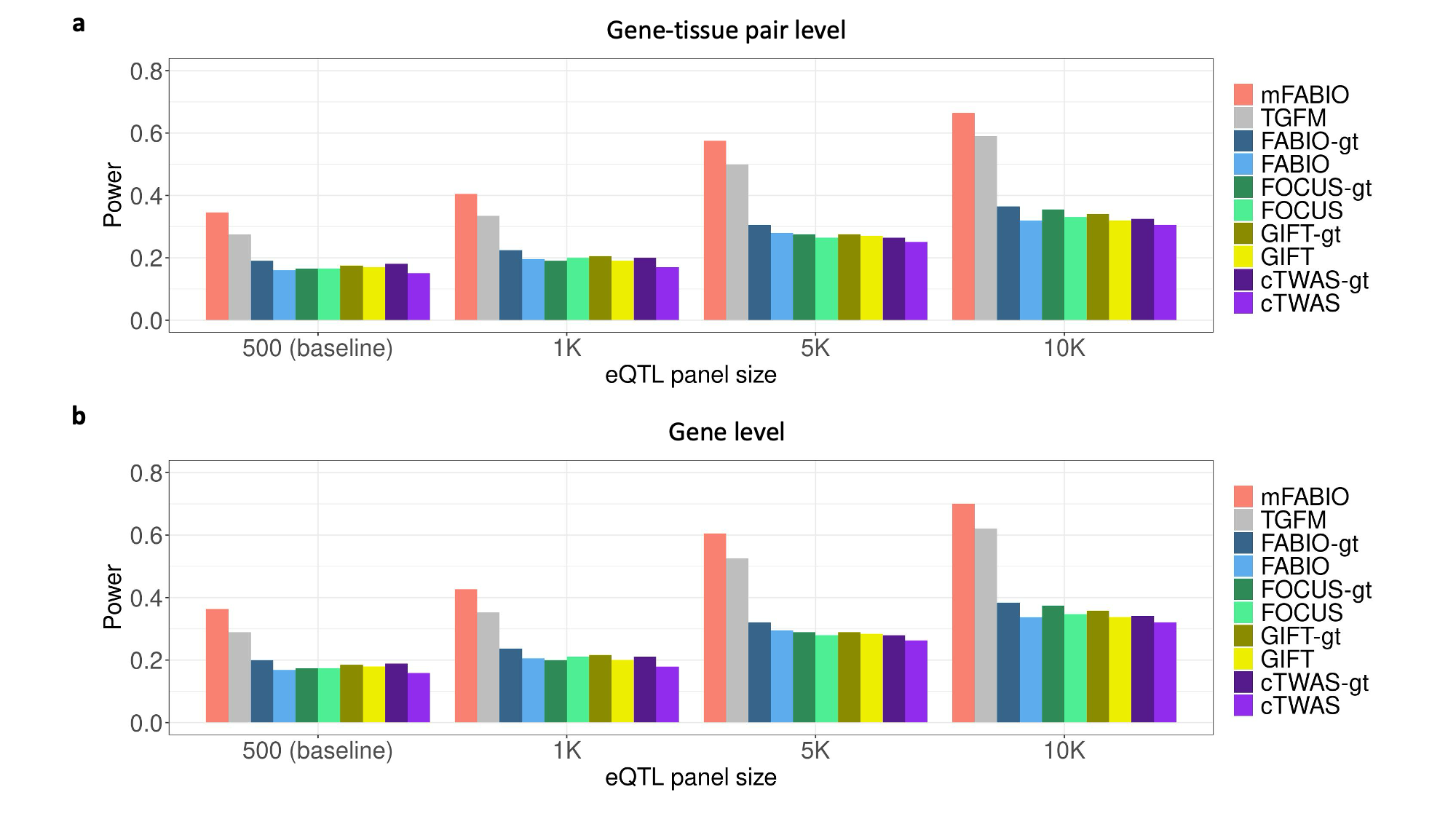

Supplement: S7 Fig — (a) Results at gene-tissue pair level. (b) Results at gene level. (TIFF) [file pgen.1012157.s007.tiff]

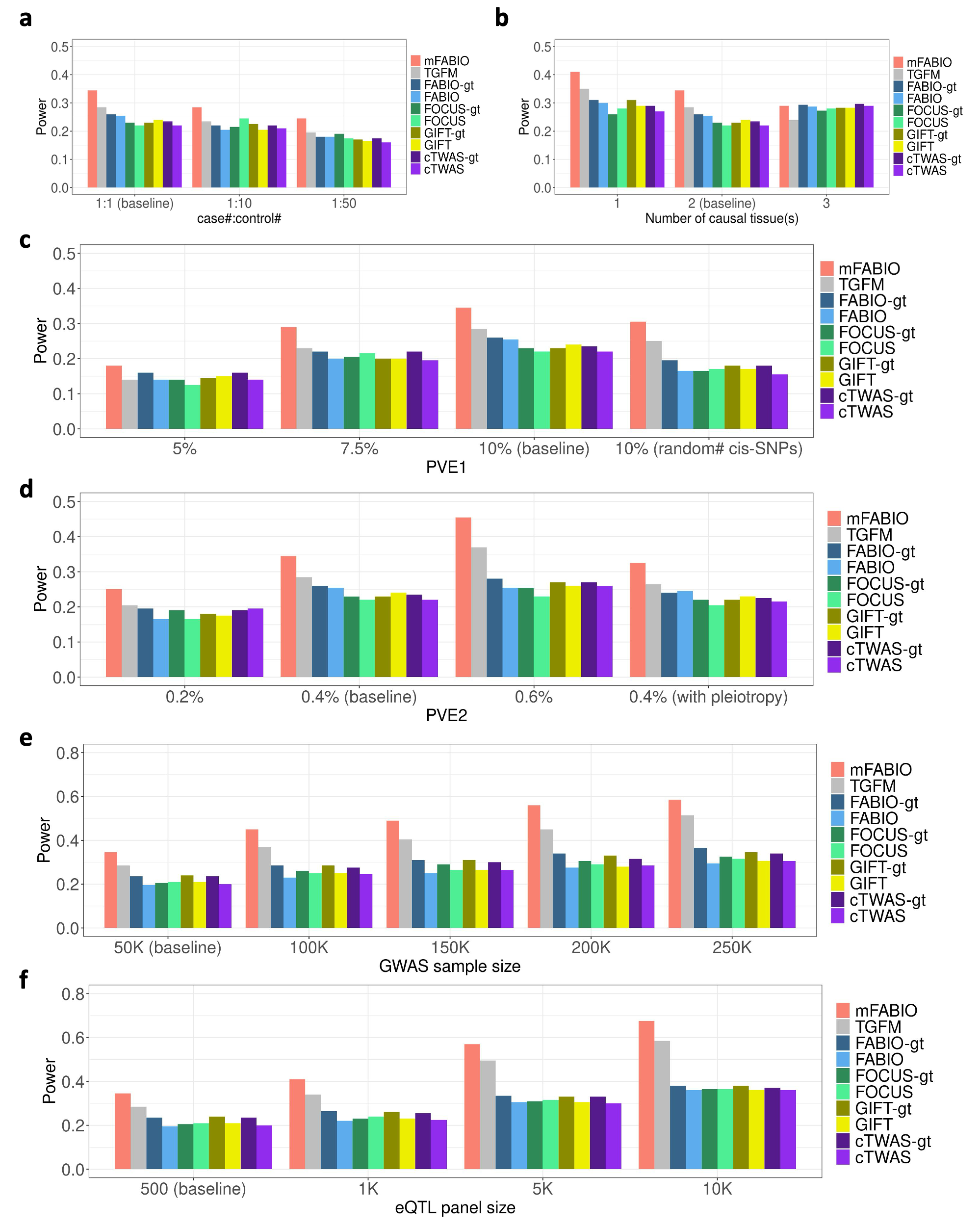

Supplement: S8 Fig — We calculated the power under an estimated FDR threshold of 0.05 to evaluate the performance of the methods in different simulation settings: (a) different case:control ratios; (b) different numbers of causal tissue(s); (c) different proportions of gene expression variance explained by genetic effects (PVE1) and different numbers of causal cis-SNPs; (d) different proportions of the phenotype’s variance explained by causal gene-tissue pairs (PVE2); (e) different GWAS sample sizes; (f) different eQTL sample sizes. (TIFF) [file pgen.1012157.s008.tiff]

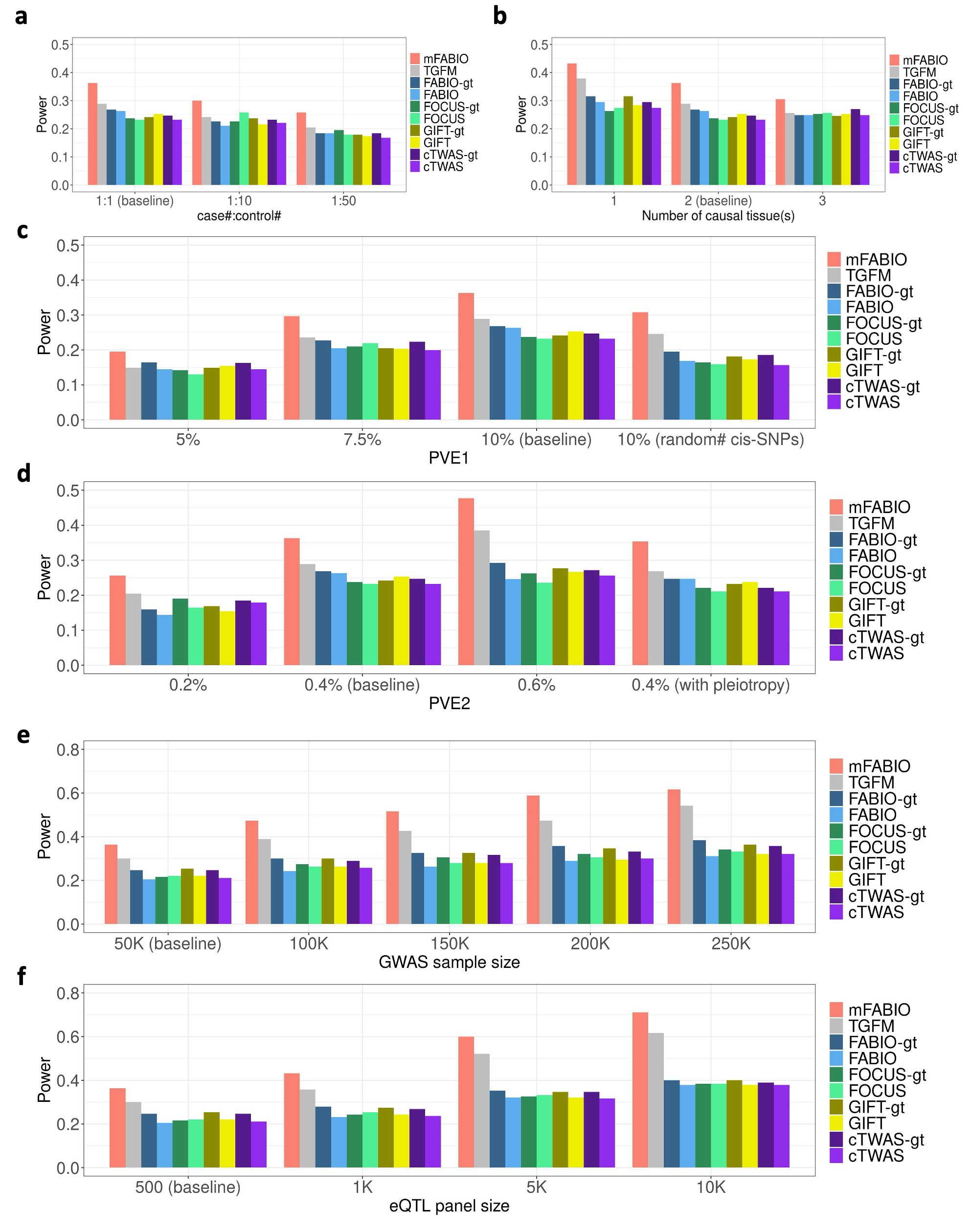

Supplement: S9 Fig — We calculated the power under an estimated FDR threshold of 0.05 to evaluate the performance of the methods in different simulation settings: (a) different case:control ratios; (b) different numbers of causal tissue(s); (c) different proportions of gene expression variance explained by genetic effects (PVE1) and different numbers of causal cis-SNPs; (d) different proportions of the phenotype’s variance explained by causal gene-tissue pairs (PVE2); (e) different GWAS sample sizes; (f) different eQTL sample sizes. (TIFF) [file pgen.1012157.s009.tiff]

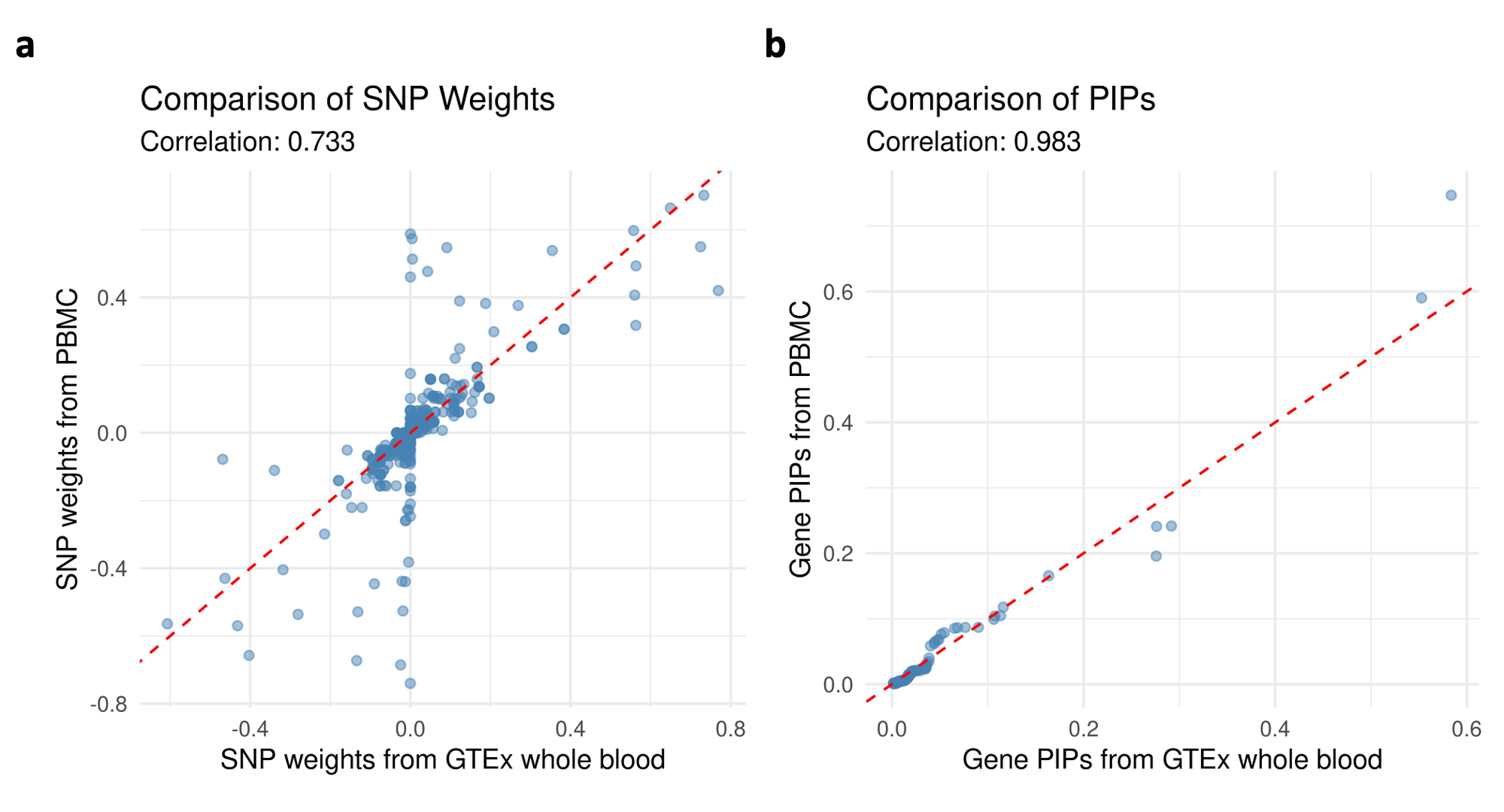

Supplement: S10 Fig — (b) Scatter plot of comparing gene PIPs of mFABIO using the PBMC eQTL panel against using the GTEx whole blood eQTL panel. (TIFF) [file pgen.1012157.s010.tiff]
