# Supplementary material for: mFABIO: An integrative multi-tissue TWAS fine-mapping approach to prioritize potentially causal genes and tissues underlying binary traits
Source: PLoS Genet. 2026 May 27;22(5):e1012157. doi: 10.1371/journal.pgen.1012157 (PMC13225670; doi:10.1371/journal.pgen.1012157)
Supplement: S1 Text — (DOCX) [file pgen.1012157.s011.docx]

**Variational inference overview**

As illustrated in the main text, we aim to minimize the Kullback-Leibler (KL) divergence between the approximate posterior and the actual posterior. Here we denote all the parameters in our model as $\boldsymbol{\Theta}\boldsymbol{=(}\mu,\beta_{l},\boldsymbol{\gamma}_{l}\boldsymbol{,}\boldsymbol{\pi}_{\boldsymbol{G}},\boldsymbol{\pi}_{\boldsymbol{T|}*}\boldsymbol{,\alpha,}\boldsymbol{,}\sigma_{\alpha}^{2}\boldsymbol{,}\sigma_{\beta}^{2}\boldsymbol{)}$. Based on our model definitions in main text Equations (2)-(5), minimizing the KL divergence is equivalent to maximizing the evidence of lower bound (ELBO) defined as:

$$ELBO=E_{q}\left[ \log p\left( \boldsymbol{y,z,}\boldsymbol{\Theta} \right) \right]-E_{q}\left[ \log q\left( \boldsymbol{z,}\boldsymbol{\Theta} \right) \right]$$

where $p\left( \boldsymbol{\cdot} \right)$ denotes the prior probability and $q\left( \boldsymbol{\cdot} \right)$ denotes the approximation posterior.

For the intercept $\mu$, we assume a normal distribution: $\mu\sim N(0, \sigma_{\mu}^{2})$, where $\sigma_{\mu}^{2}\to\infty$ as a non-informative prior following the common practice in Bayesian approaches, which is equivalent to treating it as a fixed effect. For the prior of the scalar effect‐size parameter $\beta_{l}$, we assume a normal distribution: $N\left( 0\boldsymbol{,}\sigma_{\beta}^{2} \right)$, for $l=1,2,\ldots,L$, and $\sigma_{\beta}^{2}$ has a weakly informative inverse-gamma prior: $\sigma_{\beta}^{2}\sim InverseGamma(1,1)$. For the prior of $\boldsymbol{\gamma}_{l}$, which is a *p*-vector, we assume it to follow a multinomial distribution: $\boldsymbol{\gamma}_{l}\sim Mult\left( 1,\boldsymbol{\pi} \right)$, where $\boldsymbol{\pi}$ is a *p*-vector of prior probabilities that remain the same for all *L* effects. We further denote $\boldsymbol{\gamma}_{lj}$ as the causal status of *j*'th gene-tissue pair taking value 0 or 1, and its corresponding prior probability $\pi_{j}$ is determined by hyperparameters $\boldsymbol{\pi}_{\boldsymbol{G}}$ and $\boldsymbol{\pi}_{\boldsymbol{T|}*}$ following the Equation (5) in the main text. We assume symmetric Dirichlet priors for both of the hyperparameters: $\boldsymbol{\pi}_{\boldsymbol{G}}\boldsymbol{\sim}Dirichlet(1,\ldots,1)$ and $\boldsymbol{\pi}_{\boldsymbol{T|}*}\boldsymbol{\sim}Dirichlet(1,\ldots,1)$, to represent an uninformative starting belief that all genes, and all tissues within a gene, are equally likely to be selected. Therefore, the prior probability that each gene-tissue pair displays the *l*’th non-zero effect is equally to be 1/*p*. For the prior of the SNP effect sizes $\boldsymbol{\alpha}$, we assume it to follow a multivariate normal distribution: $\boldsymbol{\alpha\sim}N\boldsymbol{(0,}\sigma_{\alpha}^{2}\boldsymbol{I)}$, and $\sigma_{\alpha}^{2}$ also has a weakly informative inverse-gamma prior: $\sigma_{\alpha}^{2}\sim InverseGamma(1,1)$. The effect sizes on covariates are also treated as a fixed effect, where we assume a normal prior distribution: $\boldsymbol{\sim}N\left( \boldsymbol{0,}\sigma^{2}\boldsymbol{I}_{c} \right)$, and $\sigma^{2}\to\infty$.

With these prior specifications, we then apply an iterative algorithm to update the parameters, by identifying the optimal approximate posterior distribution $q^{*}\left( \boldsymbol{\cdot} \right)$ to maximize the ELBO. The update for any single parameter (or variable) is proportional to the exponentiated expectation of the log joint probability, taken with respect to all other parameters and variables. We will share the derivation of the approximate posterior distributions and how we update the parameters in the following sections.

### Update of latent variable $\boldsymbol{z}$

We first update the latent variable vector $\boldsymbol{z}$ by finding the optimal approximate posterior distribution $q^{*}\left( z_{i} \right)$ for each individual’s latent variable, given the current state of all other model parameters. Assuming $\log p\left( \boldsymbol{z} \right)=\prod_{i=1}^{n} p(z_{i})$following ^1^, we first isolate the terms in ELBO that are related to each $z_{i}$, and get:

$$\log q^{*}\left( z_{i} \right)\propto E_{q}\left[ \log p\left( y_{i}|z_{i} \right)+\log p\left( z_{i}|\eta_{i} \right) \right]$$

Based on the Equation (2) in the main text, $p\left( y_{i}|z_{i} \right)$ is an indicator function:

$$p\left( y_{i}|z_{i} \right)=\left\{ \begin{aligned} 1, if \left( y_{i}=1 and z_{i}\geq0 \right) or \left( y_{i}=0 and z_{i}<0 \right) \\ 0, otherwise \end{aligned} \right..$$

Therefore, it enforces the optimal approximate posterior distribution $q^{*}\left( z_{i} \right)$ to be truncated. Based on the Equation (3) in the main text, $z_{i}$ follows a prior normal distribution: $z_{i}\sim N(\eta_{i},1)$, where $\eta_{i}=\mu+{{\hat{\boldsymbol{G}}}_{i}}^{T}\boldsymbol{\beta+}{\boldsymbol{X}_{i}}^{T}\boldsymbol{\alpha+}{\boldsymbol{C}_{i}}^{T}$, and $\log p\left( z_{i}|\eta_{i} \right)=-\frac{1}{2}\log2\pi-\frac{1}{2}{(z_{i}-\eta_{i})}^{2}$. Combining these results above, only when $\left( y_{i}=1 and z_{i}\geq0 \right) or \left( y_{i}=0 and z_{i}<0 \right)$, we have:

$$\log q^{*}\left( z_{i} \right)\propto E_{q}\left[ \log1+(-\frac{1}{2}\log2\pi-\frac{1}{2}{(z_{i}-\eta_{i})}^{2}) \right]$$

$$\propto E_{q}\left[ -\frac{1}{2}\left( z_{i}-\eta_{i} \right)^{2} \right]\propto-\frac{1}{2}({z_{i}}^{2}-2z_{i}E_{q}[\eta_{i}])\propto-\frac{1}{2}\left( z_{i}-E_{q}[\eta_{i}] \right)^{2}$$

Therefore, the optimal posterior distribution of $z_{i}$ conditional on $\boldsymbol{y}$ and other given parameter estimates is a truncated normal with mean $E_{q}[\eta_{i}]=E_{q}[\mu+{{\hat{\boldsymbol{G}}}_{i}}^{T}\boldsymbol{\beta+}{\boldsymbol{X}_{i}}^{T}\boldsymbol{\alpha+}{\boldsymbol{C}_{i}}^{T}\boldsymbol{]}$:

$$z_{i}|y_{i}=1,\mu,\boldsymbol{\beta}\boldsymbol{,}\boldsymbol{\alpha} \sim N\left( E_{q}[\eta_{i}],1 \right), truncated to [0,+\infty)$$

$$z_{i}|y_{i}=0,\mu,\boldsymbol{\beta}\boldsymbol{,}\boldsymbol{\alpha} \sim N\left( E_{q}[\eta_{i}],1 \right), truncated to (-\infty,0)$$

If $y_{i}=1$, then $z_{i}\in\left[ 0,+\infty\right)$ and we can update $E_{q}\left[ z_{i} \right]$ using the posterior mean as:

$$E_{q}\left[ z_{i} \right]=E_{q}[\eta_{i}]\boldsymbol{+}\frac{\phi\boldsymbol{(}E_{q}[\eta_{i}]\boldsymbol{)}}{\Phi(E_{q}[\eta_{i}])}$$

if $y_{i}=0$, then $z_{i}\in(-\infty,0)$ and we can update $E_{q}\left[ z_{i} \right]$ using the posterior mean as:

$$E_{q}\left[ z_{i} \right]=E_{q}[\eta_{i}]\boldsymbol{-}\frac{\phi\boldsymbol{(}E_{q}[\eta_{i}]\boldsymbol{)}}{\Phi(\boldsymbol{-}E_{q}[\eta_{i}])}$$

### where $\boldsymbol{\phi}$ is the probability density function (PDF) of the standard normal distribution, and $\boldsymbol{\Phi}$ is the cumulative distribution function (CDF) of the standard normal distribution. These updates are performed for all $\boldsymbol{i=1,\ldots,n}$, resulting in a new vector of expectations $\boldsymbol{E}_{\boldsymbol{q}}\left[ \boldsymbol{z} \right]$, which is then used to update the other parameters in the model.

### Update of $\boldsymbol{\mu}$

Next, we update those regression parameters that determine $\boldsymbol{z}$. Starting with the intercept $\mu$, which is treated as a fixed effect in the model, we update the point estimate $\tilde{\mu}$ by finding the value that maximizes the ELBO, holding all other parameters fixed. We first isolate relevant terms from the ELBO, and get:

$$\tilde{\mu}=\arg\max_{\mu} E_{q}\left[ \log p\left( \boldsymbol{z}|\mu,\boldsymbol{\beta,\alpha,} \right) \right]$$

By minimizing the sum of squared errors between the observations and their expected values, then we have:

$$\tilde{\mu}=\arg\min_{\mu} E_{q}\left[ \left\| \boldsymbol{z-(}\mu\boldsymbol{1+}\hat{\boldsymbol{G}}\boldsymbol{\beta+X\alpha+C}\boldsymbol{)} \right\|^{2} \right]$$

We take the derivative with respect to $\mu$ and set it to zero, and get the final update rule as:

$$\tilde{\mu}=\frac{1}{n}\sum_{i=1}^{n} E_{q}\left[ z_{i}-{{\hat{\boldsymbol{G}}}_{i}}^{T}\boldsymbol{\beta-}{\boldsymbol{X}_{i}}^{T}\boldsymbol{\alpha-}{\boldsymbol{C}_{i}}^{T} \right]$$

### Update of

With the normal prior assumption treating as a fix effect, we assume the optimal variational distribution as $q^{*}\left( \right)=N\boldsymbol{(}\boldsymbol{\mu}\boldsymbol{,}\boldsymbol{\Sigma}\boldsymbol{)}$. By maximizing the ELBO, the variational covariance matrix will be updated as:

$$\boldsymbol{\Sigma}{\boldsymbol{=}(\boldsymbol{C}^{T}\boldsymbol{C+}E_{q}\left[ \frac{1}{\sigma^{2}} \right]\boldsymbol{I}_{\boldsymbol{c}})}^{\boldsymbol{-}1}$$

and the variational mean vector will be updated as:

$$\boldsymbol{\mu}\boldsymbol{=}\boldsymbol{\Sigma}\boldsymbol{(}\boldsymbol{C}^{T}E_{q}\left[ \boldsymbol{z}-\mu\boldsymbol{1}_{\boldsymbol{n}}\boldsymbol{-}\hat{\boldsymbol{G}}\boldsymbol{\beta-X}\boldsymbol{\alpha} \right]\boldsymbol{)}$$

### Update of $\boldsymbol{\alpha}$ and $\boldsymbol{\sigma}_{\boldsymbol{\alpha}}^{\boldsymbol{2}}$

Given the SNP effect sizes $\boldsymbol{\alpha}$ has a multivariate normal prior: $\boldsymbol{\alpha\sim}N\boldsymbol{(0,}\sigma_{\alpha}^{2}\boldsymbol{I)}$, we assume $q^{*}\left( \boldsymbol{\alpha} \right)=N\boldsymbol{(}\boldsymbol{\mu}_{\boldsymbol{\alpha}}\boldsymbol{,}\boldsymbol{\Sigma}_{\boldsymbol{\alpha}}\boldsymbol{)}$ due to the normal-normal conjugation. We first isolate those terms in ELBO that involve the vector $\boldsymbol{\alpha}$:

$$\log q^{*}\left( \boldsymbol{\alpha} \right)\propto E_{q}\left[ \log p\left( \boldsymbol{\alpha|}\sigma_{\alpha}^{2} \right)+\log p\left( \boldsymbol{z}|\mu,\boldsymbol{\beta}\boldsymbol{,}\boldsymbol{\alpha,} \right) \right]\propto-\frac{1}{2}E_{q}\left[ \frac{1}{\sigma_{\alpha}^{2}} \right]\boldsymbol{\alpha}^{T}\boldsymbol{\alpha}+E_{q}\left[ -\frac{1}{2}\left\| \left( \boldsymbol{z}-\mu\boldsymbol{1-}\hat{\boldsymbol{G}}\boldsymbol{\beta} \right)\boldsymbol{-X}\boldsymbol{\alpha-}\boldsymbol{C} \right\|^{2} \right]$$

Then based on the log-kernel of a multivariate normal distribution, we have:

$$\boldsymbol{\Sigma}_{\boldsymbol{\alpha}}{\boldsymbol{=}(\boldsymbol{X}^{T}\boldsymbol{X+}E_{q}\left[ \frac{1}{\sigma_{\alpha}^{2}} \right]\boldsymbol{I}_{\boldsymbol{s}})}^{\boldsymbol{-}1}$$

$$\boldsymbol{\mu}_{\boldsymbol{\alpha}}\boldsymbol{=}\boldsymbol{\Sigma}_{\boldsymbol{\alpha}}\boldsymbol{(}\boldsymbol{X}^{T}E_{q}\left[ \boldsymbol{z}-\mu\boldsymbol{1}_{\boldsymbol{n}}\boldsymbol{-}\hat{\boldsymbol{G}}\boldsymbol{\beta-C} \right]\boldsymbol{)}$$

Then we focus on the update of $\sigma_{\alpha}^{2}$, given that we place an inverse-gamma prior on $\sigma_{\alpha}^{2}$: $\sigma_{\alpha}^{2}\sim InverseGamma(a_{\alpha},b_{\alpha})$, and initially $a_{\alpha}=b_{\alpha}=1$. Again, we isolate those terms in the log joint probability that involve $\sigma_{\alpha}^{2}$:

$$\log q^{*}\left( \sigma_{\alpha}^{2} \right)\propto\log p\left( \sigma_{\alpha}^{2} \right)+E_{q}\left[ \log p\left( \boldsymbol{\alpha|}\sigma_{\alpha}^{2} \right) \right]\propto-\left( a_{\alpha}+1 \right)\log\sigma_{\alpha}^{2}-\frac{b_{\alpha}}{\sigma_{\alpha}^{2}}+E_{q}\left[ -\frac{s}{2}\log2\pi-\frac{s}{2}\log\sigma_{\alpha}^{2}-\frac{1}{2\sigma_{\alpha}^{2}}\boldsymbol{\alpha}^{T}\boldsymbol{\alpha} \right]\propto\propto-\left( a_{\alpha}+1 \right)\log\sigma_{\alpha}^{2}-\frac{b_{\alpha}}{\sigma_{\alpha}^{2}}-\frac{s}{2}\log\sigma_{\alpha}^{2}-\frac{1}{2\sigma_{\alpha}^{2}}E_{q}\left[ \boldsymbol{\alpha}^{T}\boldsymbol{\alpha} \right]=-\left( a_{\alpha}+\frac{s}{2}+1 \right)\log\sigma_{\alpha}^{2}-\frac{1}{\sigma_{\alpha}^{2}}\left( b_{\alpha}+\frac{E_{q}\left[ \boldsymbol{\alpha}^{T}\boldsymbol{\alpha} \right]}{2} \right)$$

Here, $s$ is the total number of SNPs in the region of interest defined in the main text. Based on the log-kernel of an inverse-gamma distribution, the optimal posterior for $\sigma_{\alpha}^{2}$ is also an inverse-gamma distribution: $q^{*}\left( \sigma_{\alpha}^{2} \right)=InverseGamma(\tilde{a}_{\alpha},\tilde{b}_{\alpha})$, with

$$\tilde{a}_{\alpha}=a_{\alpha}+\frac{s}{2}$$

$$\tilde{b}_{\alpha}=b_{\alpha}+\frac{E_{q}\left[ \boldsymbol{\alpha}^{T}\boldsymbol{\alpha} \right]}{2}$$

where $E_{q}\left[ \boldsymbol{\alpha}^{T}\boldsymbol{\alpha} \right]=trace\left( \boldsymbol{\Sigma}_{\boldsymbol{\alpha}} \right)+{\boldsymbol{\mu}_{\boldsymbol{\alpha}}}^{T}\boldsymbol{\mu}_{\boldsymbol{\alpha}}$, based on the updated $\boldsymbol{\alpha}$ defined above.

**Update of** $\boldsymbol{\beta}_{\boldsymbol{l}}$**,** $\boldsymbol{\sigma}_{\boldsymbol{\beta}}^{\boldsymbol{2}}$**, and** $\boldsymbol{\gamma}_{\boldsymbol{l}}$

Now we move to the parameters for the Sum of Single Effects (SuSiE) prior placed on the gene-tissue effect sizes $\boldsymbol{\beta}$. As defined in the main text Equation (4), $\boldsymbol{\beta}$ is decomposed as a sum of *L* single-eﬀect components. Each single‐effect component is denoted as $\beta_{l}\boldsymbol{\gamma}_{l}$, where $\beta_{l}$ is a shared scalar effect‐size parameter following a normal prior $N\left( 0\boldsymbol{,}\sigma_{\beta}^{2} \right)$, for $l=1,2,\ldots,L$; and $\boldsymbol{\gamma}_{l}$ is a *p*-vector of binary indicators to indicate which gene-tissue pair is selected by the *l*’th component. We assume a shared prior distribution $\boldsymbol{\gamma}_{l}\sim Multinomial\left( 1,\boldsymbol{\pi} \right)$, for $l=1,2,\ldots,L$, allowing each single effect to select a specific column of $\hat{\boldsymbol{G}}$. For each of the *L* components, we update its selection probabilities with $q^{*}\left( \boldsymbol{\gamma}_{l} \right)$ and its effect size distribution with $q^{*}\left( \beta_{l} \right)$. After all *L* components have been updated, we update the shared effect size variance with $q^{*}\left( \sigma_{\beta}^{2} \right)$.

For each component *l*, we first consider a residual vector $\boldsymbol{r}_{l}$​, which serves as the target signal for the *l*’th component to explain and is denoted as:

$$\boldsymbol{r}_{l}:=\boldsymbol{z}-\mu\boldsymbol{1}_{\boldsymbol{n}}\boldsymbol{-}\boldsymbol{X}\boldsymbol{\alpha-}\sum_{l^{'}\neq l} \beta_{l^{'}}\boldsymbol{\gamma}_{l^{'}}$$

Given the conjugate prior distribution $\boldsymbol{\gamma}_{l}\sim Multinomial\left( 1,\boldsymbol{\pi} \right)$, $q^{*}\left( \boldsymbol{\gamma}_{l} \right)$ is also a multinomial distribution with one trial, denoted as $q^{*}\left( \boldsymbol{\gamma}_{l} \right)=Multinomial\left( 1,{\tilde{\boldsymbol{\pi}}}_{l} \right)$, and ${\tilde{\boldsymbol{\pi}}}_{l}\boldsymbol{=(}\tilde{\pi}_{l1},\ldots,\tilde{\pi}_{lp}\boldsymbol{)}$ for the total of *p* gene-tissue pairs. Then we have:

$$\log\tilde{\pi}_{lj}\propto E_{q}\left[ \log p\left( \boldsymbol{z|}\mu\boldsymbol{,}\boldsymbol{\alpha,}\beta_{l},\boldsymbol{\gamma}_{lj}\boldsymbol{=}1 \right) \right]+E_{q}\left[ \log p\left( \gamma_{lj}\boldsymbol{=}1 \right) \right]$$

Here, $E_{q}\left[ \log p\left( \boldsymbol{z|}\mu\boldsymbol{,}\boldsymbol{\alpha,}\beta_{l},\gamma_{lj}\boldsymbol{=}1 \right) \right]$ represents the log Bayes factor, for including the *j*’th gene-tissue pair in a single-variable regression model for the residual $\boldsymbol{r}_{l}$. It quantifies the evidence from the data in favor of a model where the *j*’th gene-tissue pair has an effect, versus a null model with only noise, and we denote it as $\log{BF}_{j}$. Here we denote the null model as ${model}_{0}: \boldsymbol{r}_{l}\boldsymbol{=\epsilon, \epsilon\sim}N\boldsymbol{(0,}\boldsymbol{I}_{n}\boldsymbol{)}$, and the alternative model as ${model}_{j}: \boldsymbol{r}_{l}\boldsymbol{=}{\hat{\boldsymbol{G}}}_{,j}\beta_{l}\boldsymbol{+\epsilon, \epsilon\sim}N\boldsymbol{(0,}\boldsymbol{I}_{n}\boldsymbol{)}$, where ${\hat{\boldsymbol{G}}}_{,j}$ is the *j*’th column of $\hat{\boldsymbol{G}}$. We further denote that when $\boldsymbol{\gamma}_{lj}\boldsymbol{=}1$, the optimal posterior distribution for $\beta_{l}$ as $q^{*}\left( \beta_{l}| \gamma_{lj}\boldsymbol{=}1 \right)=N(\mu_{\beta_{l|j}}\boldsymbol{,}\sigma_{\beta_{l|j}}^{2})$, given the normal-conjugate prior on $\beta_{l}$. We have:

$$\log q^{*}\left( \beta_{l}| \gamma_{lj}\boldsymbol{=}1 \right)\propto\log p\left( {\boldsymbol{r}_{l}|\beta}_{l} \right)+\log p\left( \beta_{l} \right)\propto\left( -\frac{n}{2}\log2\pi-\frac{1}{2}\left\| \boldsymbol{r}_{l}\boldsymbol{-}{\hat{\boldsymbol{G}}}_{,j}\beta_{l} \right\|^{2} \right)+(-\frac{\beta_{l}^{2}}{2\sigma_{\beta}^{2}})\propto\left( {{\hat{\boldsymbol{G}}}_{,j}}^{T}\boldsymbol{r}_{l} \right)\beta_{l}-\frac{1}{2}\left( {{\hat{\boldsymbol{G}}}_{,j}}^{T}{\hat{\boldsymbol{G}}}_{,j}+\frac{1}{\sigma_{\beta}^{2}} \right)\beta_{l}^{2}$$

Based on the log-kernel of the normal distribution, we solve $\mu_{\beta_{l|j}}$ and $\sigma_{\beta_{l|j}}^{2}$ as:

$$\sigma_{\beta_{l|j}}^{2}=\left( {{\hat{\boldsymbol{G}}}_{,j}}^{T}{\hat{\boldsymbol{G}}}_{,j}+\frac{1}{\sigma_{\beta}^{2}} \right)^{-1}$$

$$\mu_{\beta_{l|j}}\boldsymbol{=}\sigma_{\beta_{l|j}}^{2}\left( {{\hat{\boldsymbol{G}}}_{,j}}^{T}\boldsymbol{r}_{l} \right)$$

Then we have the log Bayes factor as:

$$\log{BF}_{j}=\log p\left( \boldsymbol{r}_{l}\boldsymbol{|}{model}_{j} \right)-\log p\left( \boldsymbol{r}_{l}\boldsymbol{|}{model}_{0} \right)=\log p\left( \boldsymbol{r}_{l}\boldsymbol{|}{\beta_{l},model}_{j} \right)+\log p\left( {\beta_{l}|model}_{j} \right)-\log p\left( \beta_{l}\boldsymbol{|}{\boldsymbol{r}_{l},model}_{j} \right)-\log p\left( \boldsymbol{r}_{l}\boldsymbol{|}{model}_{0} \right)=\left( -\frac{n}{2}\log2\pi-\frac{1}{2}\left\| \boldsymbol{r}_{l}\boldsymbol{-}{\hat{\boldsymbol{G}}}_{,j}\mu_{\beta_{l|j}} \right\|^{2} \right)+\left( -\frac{1}{2}\log2\pi\sigma_{\beta}^{2}-\frac{\mu_{\beta_{l|j}}^{2}}{2\sigma_{\beta}^{2}} \right)-\left( -\frac{1}{2}\log2\pi\sigma_{\beta_{l|j}}^{2} \right)-\left( -\frac{n}{2}\log2\pi-\frac{1}{2}{\boldsymbol{r}_{l}}^{T}\boldsymbol{r}_{l} \right)=\mu_{\beta_{l|j}}{{\hat{\boldsymbol{G}}}_{,j}}^{T}\boldsymbol{r}_{l}\boldsymbol{-}\frac{1}{2}\mu_{\beta_{l|j}}^{2}{{\hat{\boldsymbol{G}}}_{,j}}^{T}{\hat{\boldsymbol{G}}}_{,j}\boldsymbol{-}\frac{1}{2}\log\sigma_{\beta}^{2}-\frac{\mu_{\beta_{l|j}}^{2}}{2\sigma_{\beta}^{2}}+\frac{1}{2}\log\sigma_{\beta_{l|j}}^{2}=\frac{\mu_{\beta_{l|j}}^{2}}{2\sigma_{\beta_{l|j}}^{2}}+\frac{1}{2}\log\sigma_{\beta_{l|j}}^{2}\boldsymbol{-}\frac{1}{2}\log\sigma_{\beta}^{2}$$

For $E_{q}\left[ \log p\left( \boldsymbol{\gamma}_{lj}\boldsymbol{=}1 \right) \right]$, it depends on the hierarchical priors we introduced in the main text Equation (5). Following the same annotations in the main text, we assume that the *j*’th gene-tissue pair correspond to the specific pair of the *k*’th unique tissue and the *m*’th unique gene. Then we have:

$$E_{q}\left[ \log p\left( \boldsymbol{\gamma}_{lj}\boldsymbol{=}1 \right) \right]=E_{q}\left[ \log p\left( \pi_{g_{m}} \right) \right]+E_{q}\left[ \log p\left( \pi_{t_{k}|g_{m}} \right) \right]$$

We have Dirichlet priors denoted as:

$$\boldsymbol{\pi}_{\boldsymbol{G}}\boldsymbol{\sim}Dirichlet(\delta_{g_{1}},\ldots,\delta_{g_{M}})$$

$$\boldsymbol{\pi}_{\boldsymbol{T|}g_{m}}\boldsymbol{\sim}Dirichlet(\delta_{t_{1}|g_{m}},\ldots,\delta_{t_{K}|g_{m}})$$

And Dirichlet distribution is the conjugate prior distribution of the multinomial distribution, so both $q^{*}(\boldsymbol{\pi}_{\boldsymbol{G}})$ and $q^{*}(\boldsymbol{\pi}_{\boldsymbol{T|}*})$ are also Dirichlet distributions, and we denote them as:

$$q^{*}\left( \boldsymbol{\pi}_{\boldsymbol{G}} \right)=Dirichlet(\tilde{\delta}_{g_{1}},\ldots,\tilde{\delta}_{g_{M}})$$

$$q^{*}\left( \boldsymbol{\pi}_{\boldsymbol{T|}g_{m}} \right)=Dirichlet(\tilde{\delta}_{t_{1}|g_{m}},\ldots,\tilde{\delta}_{t_{K}|g_{m}})$$

It is also known that if a probability vector $\boldsymbol{\theta}=(\theta_{1},\ldots,\theta_{N})$ follows a Dirichlet distribution: $\boldsymbol{\theta\sim}Dirichlet(\delta_{1},\ldots,\delta_{N})$, then the expectation of the logarithm of its component $\theta_{i}$ ($i\in\left\{ 1,2,\ldots,N \right\}$) can be calculated as:

$$E\left[ \log\theta_{i} \right]=\psi\left( \delta_{i} \right)-\psi\left( \sum_{i=1}^{N} \delta_{i} \right)$$

where $\psi$ is the digamma function. Then we have:

$$E_{q}\left[ \log p\left( \gamma_{lj}\boldsymbol{=}1 \right) \right]=E_{q}\left[ \log p\left( \pi_{g_{m}} \right) \right]+E_{q}\left[ \log p\left( \pi_{t_{k}|g_{m}} \right) \right]=\psi\left( \tilde{\delta}_{g_{m}} \right)-\psi\left( \sum_{i=1}^{M} \tilde{\delta}_{g_{i}} \right)+\psi\left( \tilde{\delta}_{t_{k}|g_{m}} \right)-\psi\left( \sum_{j=1}^{K} \tilde{\delta}_{t_{j}|g_{m}} \right)$$

Combining the calculation of $E_{q}\left[ \log p\left( \boldsymbol{z|}\mu\boldsymbol{,}\boldsymbol{\alpha,}\beta_{l},\gamma_{lj}=1 \right) \right]$ and $E_{q}\left[ \log p\left( \gamma_{lj}\boldsymbol{=}1 \right) \right]$, now we have:

$$\log{\tilde{\boldsymbol{\pi}}}_{lj}\propto\log{BF}_{j}+E_{q}\left[ \log p\left( \gamma_{lj}\boldsymbol{=}1 \right) \right]=\frac{\mu_{\beta_{l|j}}^{2}}{2\sigma_{\beta_{l|j}}^{2}}+\frac{1}{2}\log\sigma_{\beta_{l|j}}^{2}\boldsymbol{-}\frac{1}{2}\log\sigma_{\beta}^{2}+\psi\left( \tilde{\delta}_{g_{m}} \right)-\psi\left( \sum_{i=1}^{M} \tilde{\delta}_{g_{i}} \right)+\psi\left( \tilde{\delta}_{t_{k}|g_{m}} \right)-\psi\left( \sum_{j=1}^{K} \tilde{\delta}_{t_{j}|g_{m}} \right)$$

After calculating $\log{\tilde{\boldsymbol{\pi}}}_{lj}$​ for all $j=1,\ldots,p$, we normalize them to get the final probabilities ${\tilde{\boldsymbol{\pi}}}_{l}\boldsymbol{=(}\tilde{\pi}_{l1},\ldots,\tilde{\pi}_{lp}\boldsymbol{)}$, as the update of $q^{*}\left( \boldsymbol{\gamma}_{l} \right)$.

Then we update the approximate posterior distribution for the effect size $\beta_{l}$​ with $q^{*}\left( \beta_{l} \right)$. Given the prior: $\beta_{l}\sim N(0,\sigma_{\beta}^{2})$ and the normal-normal conjugation, we assume $q^{*}\left( \beta_{l} \right)=N(\tilde{\mu}_{\beta_{l}},\tilde{\sigma}_{\beta_{l}}^{2})$. As illustrated earlier, when the *j*’th gene-tissue pair is selected as the *l*’th effect component, we have $q^{*}\left( \beta_{l}| \boldsymbol{\gamma}_{lj}\boldsymbol{=}1 \right)=N(\mu_{\beta_{l|j}}\boldsymbol{,}\sigma_{\beta_{l|j}}^{2})$, so the posterior $q^{*}\left( \beta_{l} \right)$ is the average of all these conditional posteriors, weighted by the probability of each selection $\tilde{\pi}_{lj}$ for $j=1,\ldots,p$. Therefore,

$$\tilde{\mu}_{\beta_{l}}=\sum_{j=1}^{p} \tilde{\pi}_{lj}\mu_{\beta_{l|j}}$$

$$\tilde{\sigma}_{\beta_{l}}^{2}=\sum_{j=1}^{p} \tilde{\pi}_{lj}\sigma_{\beta_{l|j}}^{2}+\left( \sum_{j=1}^{p} \tilde{\pi}_{lj}\mu_{\beta_{l|j}}^{2}-\left( \sum_{j=1}^{p} \tilde{\pi}_{lj}\mu_{\beta_{l|j}} \right)^{2} \right)=\sum_{j=1}^{p} \tilde{\pi}_{lj}\left( \mu_{\beta_{l|j}}^{2}+\sigma_{\beta_{l|j}}^{2} \right)-\tilde{\mu}_{\beta_{l}}^{2}$$

After we updated all the parameters for the *L* individual components, we update the effect size variance $\sigma_{\beta}^{2}$ that is shared among all of them. We assume an inverse-gamma prior: $\sigma_{\beta}^{2}\sim InverseGamma(a_{\beta},b_{\beta})$, and initially $a_{\beta}=b_{\beta}=1$. We isolate those terms in the log joint probability that involve $\sigma_{\beta}^{2}$:

$$\log q^{*}\left( \sigma_{\beta}^{2} \right)\propto\log p\left( \sigma_{\beta}^{2} \right)+E_{q}\left[ \sum_{l=1}^{L} \log p\left( \beta_{l}\boldsymbol{|}\sigma_{\alpha}^{2} \right) \right]\propto-\left( a_{\beta}+1 \right)\log\sigma_{\beta}^{2}-\frac{b_{\beta}}{\sigma_{\beta}^{2}}+\sum_{l=1}^{L} E_{q}\left[ -\frac{1}{2}\log2\pi-\frac{1}{2}\log\sigma_{\beta}^{2}-\frac{\beta_{l}^{2}}{2\sigma_{\beta}^{2}} \right]\propto\propto-\left( a_{\beta}+1 \right)\log\sigma_{\beta}^{2}-\frac{b_{\beta}}{\sigma_{\beta}^{2}}-\frac{L}{2}\log\sigma_{\beta}^{2}-\frac{1}{2\sigma_{\beta}^{2}}\sum_{l=1}^{L} E_{q}\left[ \beta_{l}^{2} \right]=-\left( a_{\beta}+\frac{L}{2}+1 \right)\log\sigma_{\beta}^{2}-\frac{1}{\sigma_{\beta}^{2}}\left( b_{\beta}+\frac{\sum_{l=1}^{L} E_{q}\left[ \beta_{l}^{2} \right]}{2} \right)$$

Based on the log-kernel of an inverse-gamma distribution, the optimal posterior for $\sigma_{\beta}^{2}$ is also an inverse-gamma distribution: $q^{*}\left( \sigma_{\beta}^{2} \right)=InverseGamma(\tilde{a}_{\beta},\tilde{b}_{\beta})$, with

$$\tilde{a}_{\beta}=a_{\beta}+\frac{L}{2}$$

$$\tilde{b}_{\beta}=b_{\beta}+\frac{\sum_{l=1}^{L} E_{q}\left[ \beta_{l}^{2} \right]}{2}$$

**Update of** $\boldsymbol{\pi}_{\boldsymbol{G}}$ **and** $\boldsymbol{\pi}_{\boldsymbol{T|}*}$

### Using the same annotations as the preceding section, we have the Dirichlet priors denoted as:

$$\boldsymbol{\pi}_{\boldsymbol{G}}\boldsymbol{:=(}\pi_{g_{1}},\ldots,\pi_{g_{M}}\boldsymbol{)\sim}Dirichlet(\delta_{g_{1}},\ldots,\delta_{g_{M}})$$

$$\boldsymbol{\pi}_{\boldsymbol{T|}g_{m}}\boldsymbol{:=(}\pi_{t_{1}|g_{m}},\ldots,\pi_{t_{K}|g_{m}}\boldsymbol{)\sim}Dirichlet\left( \delta_{t_{1}|g_{m}},\ldots,\delta_{t_{K}|g_{m}} \right), m=1,\ldots,M$$

We first isolate those terms in ELBO that involve $\boldsymbol{\pi}_{\boldsymbol{G}}$:

$$\log q^{*}\left( \boldsymbol{\pi}_{\boldsymbol{G}} \right)\propto\log p\left( \boldsymbol{\pi}_{\boldsymbol{G}} \right)+E_{q}\left[ \sum_{l=1}^{L} \log p\left( \boldsymbol{\gamma}_{l}\boldsymbol{|}\boldsymbol{\pi}_{\boldsymbol{G}}\boldsymbol{,}\boldsymbol{\pi}_{\boldsymbol{T|}*} \right) \right]\propto\left( \sum_{m=1}^{M} \left( \delta_{g_{m}}-1 \right)\log\pi_{g_{m}} \right)+\left( \sum_{l=1}^{L} \sum_{j=1}^{p} q\left( \gamma_{lj}=1 \right)\log(\boldsymbol{\pi}_{\boldsymbol{G}}\boldsymbol{|}\gamma_{lj}=1) \right)$$

### For the term $\sum_{\boldsymbol{l=1}}^{\boldsymbol{L}} \sum_{\boldsymbol{j=1}}^{\boldsymbol{p}} \boldsymbol{q}\left( \boldsymbol{\gamma}_{\boldsymbol{lj}}\boldsymbol{=1} \right)\log\boldsymbol{(}\boldsymbol{\pi}_{\boldsymbol{G}}\boldsymbol{|}\boldsymbol{\gamma}_{\boldsymbol{lj}}\boldsymbol{=1}\boldsymbol{)}$, we re-group the summation by gene since each gene only corresponds to partial of the total p gene-tissue pairs, then we have:

$$\log q^{*}\left( \boldsymbol{\pi}_{\boldsymbol{G}} \right)\propto\left( \sum_{m=1}^{M} \left( \delta_{g_{m}}-1 \right)\log\pi_{g_{m}} \right)+\left( \sum_{m=1}^{M} \left( \left( \sum_{l=1}^{L} \sum_{j\in g_{m} pairs} \tilde{\pi}_{lj} \right)\log\pi_{g_{m}} \right) \right)=\sum_{m=1}^{M} \left( \delta_{g_{m}}+\left( \sum_{l=1}^{L} \sum_{j\in g_{m} pairs} \tilde{\pi}_{lj} \right)-1 \right)\log\pi_{g_{m}}$$

where $g_{m} pairs$ represents the indices of all the gene-tissue pairs that contains gene $g_{m}$. And based on the log-kernel of the Dirichlet distribution, we update $\boldsymbol{\pi}_{\boldsymbol{G}}$ with:

$$q^{*}\left( \boldsymbol{\pi}_{\boldsymbol{G}} \right)=Dirichlet(\tilde{\delta}_{g_{1}},\ldots,\tilde{\delta}_{g_{M}})$$

$$\tilde{\delta}_{g_{m}}=\delta_{g_{m}}+\left( \sum_{l=1}^{L} \sum_{j\in g_{m} pairs} \tilde{\pi}_{lj} \right), m=1,\ldots,M$$

Then we isolate those terms in ELBO that involve $\boldsymbol{\pi}_{\boldsymbol{T|}*}$, and take gene $g_{m}$ as an example:

$$\log q^{*}\left( \boldsymbol{\pi}_{\boldsymbol{T|}g_{m}} \right)\propto\log p\left( \boldsymbol{\pi}_{\boldsymbol{T|}g_{m}} \right)+E_{q}\left[ \sum_{l=1}^{L} \log p\left( \boldsymbol{\gamma}_{l}\boldsymbol{|}\boldsymbol{\pi}_{\boldsymbol{G}}\boldsymbol{,}\boldsymbol{\pi}_{\boldsymbol{T|}*} \right) \right]\propto\left( \sum_{k=1}^{K} \left( \delta_{t_{k}|g_{m}}-1 \right)\log\pi_{t_{k}|g_{m}} \right)+\left( \sum_{l=1}^{L} \sum_{j=1}^{p} q\left( \gamma_{lj}=1 \right)\log(\boldsymbol{\pi}_{\boldsymbol{T|}g_{m}}\boldsymbol{|}\gamma_{lj}=1) \right)$$

For the term $\sum_{l=1}^{L} \sum_{j=1}^{p} q\left( \gamma_{lj}=1 \right)\log(\boldsymbol{\pi}_{\boldsymbol{T|}g_{m}}\boldsymbol{|}\gamma_{lj}=1)$ here, similarly we re-group the summation by tissue, then we have:

$$\log q^{*}\left( \boldsymbol{\pi}_{\boldsymbol{T|}g_{m}} \right)\propto\left( \sum_{k=1}^{K} \left( \delta_{t_{k}|g_{m}}-1 \right)\log\pi_{t_{k}|g_{m}} \right)+\left( \sum_{k=1}^{K} \left( \sum_{l=1}^{L} \sum_{j=pair \left\{ g_{m},t_{k} \right\}} \tilde{\pi}_{lj} \right)\log\pi_{t_{k}|g_{m}} \right)=\sum_{k=1}^{K} \left( \delta_{t_{k}|g_{m}}+\left( \sum_{l=1}^{L} \sum_{j=index \left\{ g_{m},t_{k} \right\}} \tilde{\pi}_{lj} \right)-1 \right)\log\pi_{t_{k}|g_{m}}$$

Where $index \left\{ g_{m},t_{k} \right\}$ represents the index of the specific gene-tissue pair $\left\{ g_{m},t_{k} \right\}$. And based on the log-kernel of the Dirichlet distribution, we update $\boldsymbol{\pi}_{\boldsymbol{T|}g_{m}}$ with:

$$q^{*}\left( \boldsymbol{\pi}_{\boldsymbol{T|}g_{m}} \right)=Dirichlet(\tilde{\delta}_{t_{1}|g_{m}},\ldots,\tilde{\delta}_{t_{K}|g_{m}})$$

$$\tilde{\delta}_{t_{k}|g_{m}}=\delta_{t_{k}|g_{m}}+\left( \sum_{l=1}^{L} \sum_{j=index \left\{ g_{m},t_{k} \right\}} \tilde{\pi}_{lj} \right), k=1,\ldots,K$$

**Summary of variational inference**

In summary, for each effect from 1 to *L*, we update the optimal posterior distributions for the parameters by maximizing the corresponding ELBO. The update steps above can be summarized in the form of Iterative Bayesian stepwise selection (IBSS) algorithm:

### Iterative Bayesian stepwise selection (IBSS) Algorithm

Required input #1: $\hat{\boldsymbol{G}}\boldsymbol{,X,y,C}(optional)$

Required input #2: Number of the non-zero effects *L,* initial estimates of hyperparameters $\sigma_{\alpha}^{2}\boldsymbol{,}\sigma_{\beta}^{2},\delta_{g_{1}},\ldots,\delta_{g_{M}}$, and $\delta_{t_{1}|g_{m}},\ldots,\delta_{t_{K}|g_{m}}$ for $m=1,\ldots,M$

1: Repeat

2: update latent variable vector $\boldsymbol{z}$

3: update regression parameters that determine $\boldsymbol{z}$: $\mu$, , $\boldsymbol{\alpha}$, and $\sigma_{\alpha}^{2}$

4: for *l* in *1*: *L* do

5: update $\boldsymbol{\gamma}_{l}$ based on the current $\boldsymbol{\pi}_{\boldsymbol{G}}$ and $\boldsymbol{\pi}_{\boldsymbol{T|}*}$

6: update $\beta_{l}$

7: end for

8: update $\sigma_{\beta}^{2}$ that is shared among all *L* components

9: update $\boldsymbol{\pi}_{\boldsymbol{G}}$ and $\boldsymbol{\pi}_{\boldsymbol{T|}*}$ based on the posterior distribution of the current $\boldsymbol{\gamma}_{l}$

10: Loop over previous steps until the convergence, where the change of ELBO between consecutive iterations is below 0.001

**Reference**

1 Held, L. & Holmes, C. C. Bayesian auxiliary variable models for binary and multinomial regression. (2006).
