# Supplementary material for: mFABIO: An integrative multi-tissue TWAS fine-mapping approach to prioritize potentially causal genes and tissues underlying binary traits
Source: PLoS Genet. 2026 May 27;22(5):e1012157. doi: 10.1371/journal.pgen.1012157 (PMC13225670; doi:10.1371/journal.pgen.1012157)
Supplement: Attachment — Submitted filename: response_letter.docx [file pgen.1012157.s013.docx]

**Comments From Reviewer #1:**

The manuscript presents a multi-tissue TWAS fine-mapping method designed for binary traits, accounting for correlations in genetically regulated expression (GReX) of genes. Simulation studies demonstrate that the method provides substantial power gains while maintaining robust control of false discovery rates.

Thank you for your positive and constructive comments. Our detailed responses to each of your comments are listed below.

Although the proposed method shows good potential, it could be further improved by more clearly addressing how to account for pleiotropic effects from SNPs.

Thank you for your comment. In response, we have revised the manuscript to clarify how pleiotropic effects from SNPs are accounted for in the model. Briefly, we denote $\boldsymbol{X}$ as an n × s genotype matrix for the n individuals, capturing s SNPs within the region of focus. In equation (1) of the revised main text, we specify

$$P\left( y_{i}=1 | {\hat{\boldsymbol{G}}}_{i},\boldsymbol{X}_{i}\boldsymbol{,}\boldsymbol{C}_{i}\boldsymbol{,\beta,\alpha,}\boldsymbol{\xi} \right)=1-P\left( y_{i}=0 | {\hat{\boldsymbol{G}}}_{i},\boldsymbol{X}_{i}\boldsymbol{,}\boldsymbol{C}_{i}\boldsymbol{,\beta,\alpha,}\boldsymbol{\xi} \right)$$

$=\Phi\left( \mu+{{\hat{\boldsymbol{G}}}_{i}}^{T}\boldsymbol{\beta+}{\boldsymbol{X}_{i}}^{T}\boldsymbol{\alpha+}{\boldsymbol{C}_{i}}^{T}\boldsymbol{\xi} \right)\left( i=1,\cdots,n \right).$	(1)

Here, $\boldsymbol{X}_{i}$ is an *s*-vector of genotypes for the *i*’th individual (i.e. the *i*’th row of $\boldsymbol{X}$); and $\boldsymbol{\alpha}$ is an *s*-vector of corresponding SNP pleiotropic effect sizes, representing the horizontal pleiotropic effects that capture SNP influences on the trait not mediated through gene expression. To account for the correlation structure among SNPs, we model the pleiotropic genetic effects $\boldsymbol{\alpha}$ using a Bayesian ridge regression framework. In particular, we place an independent and identically distributed (i.i.d.) Gaussian prior on the SNP-effect vector $\boldsymbol{\alpha}$ across the s SNPs:

$\boldsymbol{\alpha\sim}N\boldsymbol{(0,}\sigma_{\alpha}^{2}\boldsymbol{I}_{s}\boldsymbol{)}$, (4)

where $\sigma_{\alpha}^{2}$ follows a conjugate, weakly informative inverse-gamma prior, $\sigma_{\alpha}^{2}\sim IG(1,1)$. By imposing a ridge penalty on the pleiotropic effects, the model robustly accommodates the high multicollinearity inherent in genotype data. Moreover, explicit control of pleiotropic effects ensures that the variable selection mechanism (via SuSiE) remains focused on the primary features of interest, namely the gene-tissue pairs.

These details have been summarized and incorporated into the revised Materials and Methods section (**lines 144-157 on pages 8-9**) to clarify how pleiotropic effects from SNPs are modeled in our framework.

In addition, it seems how to account for the correlation among GReX of genes in a LD region is not clearly described in the fine mapping.

Thank you for your comment. Our framework account for correlation among GReX of genes within an LD region through both joint modeling of all GReX variables in the region and the Sum of Single Effects (SuSiE) prior structure placed on the gene-tissue effects $\boldsymbol{\beta}$. Specifically, all GReX are assembled into a *n* by *p* design matrix $\hat{\boldsymbol{G}}$ (*n* is the number of individuals, *p* is the number of gene-tissue pairs), with ${{\hat{\boldsymbol{G}}}_{i}}^{T}$denoting the row vector of GReX values for individual *i* in equation (1) (as described in our response to the previous comment). By jointly modeling the effects of all GReX in the region, the correlation structure among GReX induced by LD is directly incorporated into the likelihood. Additionally, unlike standard regression or simple shrinkage methods, the SuSiE prior decomposes the total genetic signal as a sum of *L* single-eﬀect components: $\boldsymbol{\beta}:=\sum_{l=1}^{L} \beta_{l}\boldsymbol{\gamma}_{l}$, where each component $\beta_{l}\boldsymbol{\gamma}_{l}$ is constrained to select exactly one GReX with a non-zero effect within the LD region. This constraint induces explicit competition among correlated GReX for inclusion in the model. During variational inference, the posterior probability for each gene-tissue pair is evaluated conditional on the effects of all other components, including the GReX of other pairs. Consequently, if two gene-tissue pairs are highly correlated but one provides a better explanation of the phenotype, the model assigns most of the probability mass to that pair, effectively explaining away the signal from the other. As a result, the resulting posterior inclusion probabilities (PIPs) reflect the unique explanatory contribution of each gene-tissue pair, while properly accounting for correlation among GReX driven by LD.

These details have been summarized and incorporated into the revised Materials and Methods section (**lines 167-193 on pages 9-11**) to clarify how our models account for the correlation among GReX of gene-tissue pairs within an LD region.

Minor comments.

It would be helpful add page number and line numbers.

Thank you for the comment on the format. We added the page numbers and line numbers in the revised manuscript.

In model (1)-(4), do the models include all SNPs in a region under consideration.

Yes, all SNPs in a LD region are under consideration in our models. We incorporate the full genotype matrix of all SNPs as input, which is denoted as ***X*** in equations (1)-(4). We have clarified this point in the revised Materials and Methods section (**lines 119-120 on page 7**).

It would be helpful to explain how to account for the correlation among SNPs or how to select SNPs in a region to be included in a model.

Thank you for your comment. We account for correlation among SNPs in the model in two complementary ways, analogous to how correlation among GReX is handled. First, we include the genotypes of all SNPs within an LD region jointly as an input matrix ***X*** in the model. By modeling all SNPs simultaneously, the correlation structure induced by LD is directly incorporated into the likelihood. Second, we model the genetic effects of SNPs, denoted by $\boldsymbol{\alpha}$ in equations (1)-(4), using a Bayesian ridge regression framework. The Gaussian prior on $\boldsymbol{\alpha}$ induces a ridge penalty that robustly handles the high multicollinearity inherent in genotype data.

Following your suggestions, we have summarized and incorporated these explanations into the revised Materials and Methods section (**lines 144-157 on pages 8-9**) to clarify how our model accounts for correlation among SNPs.

Is it reasonable to assume the effects of SNPs in a region follow iid as in the models?

Thank you for your comment. The iid normal assumption on the *prior* SNP effect sizes is standard in polygenic modeling within the statistical genetics literature [Yang, et al., 2010]. Although SNP effects are assumed to be independent *a priori*, their posteriors estimates need not be independent. In particular, the likelihood incorporates the correlation structure among SNPs induced by linkage disequilibrium, so the posterior distributions of SNP effects can be correlated and can reflect shared or similar effect patterns when supported by the data. These points have been summarized and incorporated into the revised Materials and Methods section (**lines 150-155 on page 9**).

It seems the function in model (7) do not depend on observed data G_hat and X. It would be helpful to explain how to use the observed data in the parameter estimation.

Thank you for your comment. In the original presentation, we followed variational inference conventions and omitted the explicit dependence on the observed data $(\hat{\boldsymbol{G}}\boldsymbol{,X,C,y})$ in equation (7). Specifically, equation (7) describes the factorization of the approximate posterior distribution $q\left( \boldsymbol{z,}\mu,\boldsymbol{\beta,\alpha,}\boldsymbol{\xi} \right)$:

$q\left( \boldsymbol{z,}\mu,\boldsymbol{\beta,\alpha,}\boldsymbol{\xi} \right)=q(\boldsymbol{z})\times q(\mu)\times q(\boldsymbol{\alpha})\times q(\boldsymbol{\xi} )\times\prod_{l=1}^{L} q\left( \beta_{l} \right)q(\boldsymbol{\gamma}_{l})$ $=\left( \prod_{i=1}^{n} q(z_{i}) \right)\times q(\mu)\times q(\boldsymbol{\alpha})\times q(\boldsymbol{\xi} )\times\left( \prod_{l=1}^{L} q(\beta_{l})q(\boldsymbol{\gamma}_{l}|\boldsymbol{\pi}_{\boldsymbol{G}},\boldsymbol{\pi}_{\boldsymbol{T|}*})q(\boldsymbol{\pi}_{\boldsymbol{G}})\left( \prod_{*=g_{1}}^{g_{M}} q(\boldsymbol{\pi}_{\boldsymbol{T|}*}) \right) \right)$ (7)

where $\boldsymbol{z}$ denotes latent variables and $\left( \mu,\boldsymbol{\beta,\alpha,}\boldsymbol{\xi} \right)$ are model parameters. As an approximate posterior distribution, $q\left( \boldsymbol{z,}\mu,\boldsymbol{\beta,\alpha,}\boldsymbol{\xi} \right)$, as well as each of its factorized components on the right-hand side of the equation, depends on the observed data $(\hat{\boldsymbol{G}}\boldsymbol{,X,C,y})$. This dependency is also explicitly ensured through optimization of the evidence lower bound (ELBO), which is itself a function of the observed data. Thus, although the dependence on the observed data is not shown explicitly in the notation, it is fully incorporated through the likelihood term during variational optimization. Following your comment, we have added clarifying sentences to the revised Materials and Methods section (**lines 238-244 on page 13**) to explicitly state that equation (7) depends on all observed data, with this dependence omitted solely for notational simplicity.

In the simulation studies, when generating a binary trait via latent variables, only contribution from genes are considered; how about contribution from SNPs? Is it possible some SNP affect gene expression and also have effects on the latent variables not through the genes.

Thank you for your comment. Following your suggestion, we have included a new simulation scenario in which SNPs influence the latent variable directly, bypassing gene expression. Specifically, following [Zhao, et al., 2024], we assumed a total phenotypic heritability of 0.1. We set the variance explained by GReX effects to 4% same as our baseline setting, distributed as 0.4% per pair across 10 causal gene-tissue pairs. Consequently, the remaining genetic variance (6%) was assigned to SNP horizontal pleiotropy. We found that the power of each method decreased slightly due to confounding from the pleiotropic effects. However, mFABIO still outperformed other methods at both the gene-tissue pair level and the gene level. The results are shown in Supplementary Figure S5, with details incorporated in the Materials and Methods section (**lines 349-355 on pages 17-18**), and the Results section (**lines 615-618 on page 31**). The corresponding figures are also attached below for your reference.

**Fig S5. Power of different methods under the true FDR threshold of 0.05 across different PVE2**. (a) When pleiotropic effects were involved, the power of each method decreased slightly with mFABIO still outperforming other methods at the gene-tissue pair level. (b) Same observations at gene level.

**Comments From Reviewer #2:**

The authors present mFABIO, a multi-tissue TWAS fine-mapping framework specifically developed for binary outcomes. Methodologically, mFABIO (i) models a multi-gene, multi-tissue GReX design within genomic loci, (ii) employs a probit latent-liability model suitable for case-control traits, and (iii) adopts a SuSiE-style sum-of-single-effects prior with a hierarchical Dirichlet structure to estimate posterior inclusion probabilities (PIPs) at both the gene and gene-tissue levels. The use of variational Bayes ensures computational scalability. Through extensive simulations and applications to six binary disease traits from the UK Biobank, the method demonstrates improved calibration and higher power compared with single-tissue fine-mapping tools, as well as performance gains over TGFM, while producing more concise and biologically supported candidate sets. The manuscript is clearly written and technically sound. I have the following comments:

Thank you for your positive and constructive comments. Our detailed responses to your major and other comments are listed below.

1. Equations (1) and (3) incorporate gene-tissue effect sizes and horizontal pleiotropic effects, thereby capturing SNP influences not mediated through gene expression. Could the authors clarify whether mFABIO can also account for covariates such as age, sex, and principal components? Are these effects explicitly modeled within the probit framework, or should users adjust for them externally before applying mFABIO?

Thank you for your comment. While the previous version of our model did not explicitly account for covariates, we have now extended both the model and software implementation to handle them within mFABIO framework. Specifically, we included an additional term for covariates in equation (1) as follows:

$$P\left( y_{i}=1 | {\hat{\boldsymbol{G}}}_{i},\boldsymbol{X}_{i}\boldsymbol{,}\boldsymbol{C}_{i}\boldsymbol{,\beta,\alpha,}\boldsymbol{\xi} \right)=1-P\left( y_{i}=0 | {\hat{\boldsymbol{G}}}_{i},\boldsymbol{X}_{i}\boldsymbol{,}\boldsymbol{C}_{i}\boldsymbol{,\beta,\alpha,}\boldsymbol{\xi} \right)$$

$=\Phi\left( \mu+{{\hat{\boldsymbol{G}}}_{i}}^{T}\boldsymbol{\beta+}{\boldsymbol{X}_{i}}^{T}\boldsymbol{\alpha+}{\boldsymbol{C}_{i}}^{T}\boldsymbol{\xi} \right)\left( i=1,\cdots,n \right)$	(1)

Here, $\boldsymbol{C}$ is an *n* × *c* covariate matrix for *n* individuals and *c* covariates like age, sex, and principal components; $\boldsymbol{C}_{i}$ is a *c*-vector of covariates for the *i*’th individual (i.e. the *i*’th row of $\boldsymbol{C}$); $\boldsymbol{\xi}$ is a *c*-vector of corresponding effect sizes for covariates. All other notations remain as previously defined: $y_{i}\in\left\{ 0,1 \right\}$ is the binary trait for the *i*’th individual; ${\hat{\boldsymbol{G}}}_{i}$ is a *p*-vector of GReX for *p* gene-tissue pairs for the *i*’th individual (i.e. the *i*’th row of $\hat{\boldsymbol{G}}$); $\boldsymbol{\beta}$ is a *p*-vector of corresponding effect sizes for the gene-tissue pairs; $\boldsymbol{X}_{i}$ is an *s*-vector of genotypes for the *i*’th individual (i.e. the *i*’th row of $\boldsymbol{X}$); $\boldsymbol{\alpha}$ is an *s*-vector of corresponding effect sizes for SNPs, also known as the horizontal pleiotropic effects, capturing the SNPs’ effects on the trait not mediated through gene expression; $\mu$ is a scalar representing the intercept; and $\Phi$ is the cumulative distribution function (CDF) of the standard normal distribution.

To evaluate the updated model, we applied it to the same UK Biobank (UKBB) asthma dataset, this time including sex and the top 10 genotype principal components as covariates. We observed minimal differences between the models with and without covariates (57 vs. 58 identified genes; 41 vs. 41 identified gene-tissue pairs). We have included this alternative analysis in the revised manuscript and updated the software to allow users to apply the covariate-adjusted model in future analysis. These updates are described in the revised Materials and Methods (**lines 120-136 on pages 7-8**) and Results (**lines 665-671 on page 33**) sections. The detailed derivations provided in the Supplementary Text are also updated with the new covariates term $\boldsymbol{C}\boldsymbol{\xi}$ in the corresponding steps.

In addition, it would be interesting to discuss whether the authors plan to extend mFABIO to a mixed-effects framework in the future, which could model correlations among individuals (e.g., related samples or population structure).

We appreciate this suggestion. It is worth noting that the current model’s inclusion of the polygenic term $\boldsymbol{X\alpha}$ with a Gaussian prior on $\boldsymbol{\alpha}$ is statistically equivalent to a random effect term, which follows a multivariate normal distribution with a covariance matrix derived from the SNPs in $\boldsymbol{X}$. Consequently, our method implicitly accounts for part of the correlations among individuals arising from their cis-genotype similarities in the local genomic region. With that said, we fully agree with the reviewer that additional random effect terms could be valuable in many applications. For example, incorporating trans-genotypes or pedigree information could help capture other aspects of individual similarity. Explicitly adding such terms and extending the model to a full mixed-effects framework, unfortunately, would substantially increase computational cost. Depending on the application, assumptions on the structure of the random effects, such as low-rank approximations, may be necessary to mitigate this cost. Therefore, a formal extension to a generalized mixed-effects framework, allowing users to input arbitrary covariance matrices derived from pedigrees or other sources, would provide valuable flexibility, although the details of such an extension will require careful consideration in future work. Following your suggestion, we have added a paragraph to the Discussion section (**lines 882-890 on page 44**) outlining this as a key direction for future work.

2. In the Simulations section, the authors use 50,000 individuals of European ancestry from the UK Biobank for GWAS data and 500 distinct individuals for eQTL mapping. Given the complexity of the Bayesian inference procedure, readers would benefit from a discussion of runtime and memory usage.

Thank you for your comment. In response, we conducted a comprehensive analysis on runtime and memory usage in our real data analysis using 337,198 individuals from the UK Biobank. With using 8 CPU threads, we found that mFABIO required an average of 9.77 hours to sequentially fine-map all LD loci across a single chromosome (average 65 LD loci per chromosome). In comparison, methods relying on GWAS summary statistics required an average of 7.21 hours per chromosome to first generate the necessary input GWAS summary statistics, followed by an additional 1.02 hours for TGFM and 0.90-5.84 hours for single-tissue methods to conduct fine-mapping. Regarding memory usage, the peak memory usage for mFABIO is ~10 GB. This compares to a peak memory usage of ~16 GB for the GWAS summary statistics precomputation step required by other methods, along with 4-8 GB of memory for the subsequent fine-mapping step. We have added a sub-section detailing these benchmarking results to the Results section (**lines 812-830 on page 41**).

A comparison of computational performance and statistical power across varying GWAS sample sizes (e.g., 50K, 100K, 150K, 200K, 250K) and eQTL sample sizes (e.g., 500, 1,000, 5,000, 10,000), alongside existing methods such as TGFM, FABIO, FOCUS, GIFT, and cTWAS, would be informative for understanding the scalability, efficiency, and practical utility of mFABIO for large biobank-scale analyses.

Thank you for the suggestion to extend our simulation study to evaluate performance across a broader range of sample sizes. Accordingly, we conducted additional simulations with varying GWAS sample sizes (50K, 100K, 150K, 200K, 250K) and eQTL reference sample sizes (500, 1,000, 5,000, 10,000), while keeping all other parameters fixed at baseline settings. As expected, we observed a consistent increase in statistical power with larger GWAS and eQTL sample sizes across all evaluated methods. Notably, the relative performance ranking of the methods remained stable across these variations, and mFABIO consistently maintained superior performance compared to existing approaches in all scenarios, demonstrating robust power and FDR control regardless of sample size.

Regarding computational performance, both runtime and memory usage for mFABIO scaled linearly with GWAS sample size, consistent with the algorithmic complexity. At the maximum simulated GWAS sample size (N=250K), mFABIO required approximately 2.66 hours and 8 GB of memory to sequentially fine-map all 50 LD loci in a single replicate of simulation. In comparison, summary-statistic based methods required 1.95-3.78 hours (about 1.7 hours for GWAS summary statistics computation and 0.25-2.08 hours for fine-mapping) and 8 GB of memory for GWAS summary statistics computation following with 3-6 GB for fine-mapping. These simulation benchmarks align with the runtime and memory usage observed in our real data applications, as detailed in the response to the previous comment.

These results confirm that mFABIO can effectively leverage larger datasets and benefits from both expanded GWAS cohorts and larger eQTL reference panels, while maintaining a reasonable computational efficiency. We have summarized these findings in the Results section (**lines 620-632 on pages 31-32**) and included the corresponding performance plots as Supplementary Figures S6-S7. The corresponding figures are also attached below for your reference.

**Fig. S6** **Power of different methods under the true FDR threshold of 0.05 across different GWAS sample sizes.** (a) Bar plot showing power to prioritize causal gene-tissue pairs for different methods across increasing GWAS sample sizes (50K-250K individuals). Power increases with larger GWAS sample sizes, with mFABIO consistently achieving the highest power across scenarios. (b) Same observations at gene level.

**Fig. S7** **Power of different methods under the true FDR threshold of 0.05 across different eQTL sample sizes.** (a) Bar plot showing power to prioritize causal gene-tissue pairs for different methods across increasing eQTL sample sizes (500-10K individuals). Power increases with larger eQTL sample sizes, with mFABIO consistently achieving the highest power across scenarios. (b) Same observations at gene level.

3. Both the simulation study and the UK Biobank application rely on eQTL reference data from a single source (500 individuals for the simulations and GTEx for the real data analysis). In many practical settings, eQTL models across tissues may come from different studies or consortia (e.g., GTEx, CMC, ROSMAP, Braineac, CAGE, eQTLGen) (see Methods of doi: 10.1038/s41467-018-04558-1). It would be helpful if the authors could clarify whether mFABIO remains applicable when eQTL reference panels across tissues originate from independent cohorts. For example, does the method require harmonized LD and allele frequencies across tissues, or is it sufficient to provide SNP prediction weights for each gene-tissue pair? A short discussion on this would improve readers’ understanding of mFABIO’s robustness and generalizability.

Thank you for raising this important point regarding the use of eQTL models derived from different studies or consortia. To assess the robustness of mFABIO under this scenario, we performed an additional analysis in which one tissue-specific eQTL panel was replaced with an independent reference panel from a different study. Specifically, using the same UK Biobank (UKBB) hypertension dataset, we replaced the original GTEx whole blood eQTL panel (n = 320) with a pseudobulk PBMC eQTL panel (n = 113, SNP weights publicly available at <https://doi.org/10.7910/DVN/8UL8XB>), while keeping all other tissue models from GTEx unchanged. To ensure direct comparability to the GTEx whole blood panel, the PBMC eQTL panel was restricted to European-ancestry samples, and harmonized to the same genomic reference build and variant set used in GTEx. In addition, gene expression normalization procedures were matched to those used in GTEx, and allele alignment and variant harmonization were performed by the panel’s publisher to ensure consistency with the GTEx reference models. Eventually, it led to 628 genes modeled in both panels, comprising 538,801 SNPs.

We found that the SNP weights from the two panels (GTEx and PBMC) were reasonably highly correlated (Supplementary Figure S10a). In the fine-mapping analysis, mFABIO identified the same set of significant genes for blood tissue, and the corresponding posterior inclusion probabilities (PIPs) were also highly correlated between the two panels (Supplementary Figure S10b). These results suggest that mFABIO remains reasonably robust when eQTL reference panels originate from independent cohorts, provided that ancestry is matched and appropriate harmonization procedures are applied. We do acknowledge that, if tissues are derived from cohorts with different genetic ancestries, cross-panel integration becomes more challenging due to differences in LD structure and allele frequencies.

**Fig. S10** (a) Scatter plot of comparing SNP weights generated from the PBMC eQTL panel against those from the GTEx whole blood eQTL panel. (b) Scatter plot of comparing gene PIPs of mFABIO using the PBMC eQTL panel against using the GTEx whole blood eQTL panel.

Another related scenario arises when multiple eQTL reference panels exist for the same tissue across independent cohorts. In such cases, harmonization will be required to construct a unified set of SNP weights prior to fine-mapping. Methods such as SR-TWAS (Stacked Regression TWAS) [Parrish et al., 2024] provide effective approaches for integrating SNP weights across multiple reference panels, although they require access to individual-level eQTL training data. Because individual-level data were not publicly available for the PBMC panel used here, we were unable to explore this approach. Future extensions and validations will be possible as additional individual-level eQTL datasets from diverse studies or consortia become accessible.

Following your suggestion, we have added paragraphs to the Methods and Discussion sections (**lines 471-480 on page 23,** **lines 864-880 on pages 43-44**) summarizing the above results and clarifying the robustness and generalizability of mFABIO.

Other comments:

In Figure 1, the notation “SNP prediction weights for each of the M genes, which can be obtained from eQTL mapping cohorts across different tissues” may be slightly ambiguous. For example, the same notation (w11, …, w1S1) for gene 1 in both tissue 1 and tissue K, which could give the impression that SNP weights are shared across tissues. Clarifying that these weights are tissue-specific would avoid possible misunderstanding.

Thank you for identifying this potential ambiguity. We agree that the original notation could have been interpreted as implying shared weights across tissues. To clarify this, we have updated Figure 1 to include tissue-specific indices (superscripts) in the weight notation (e.g., $w_{T_{K}S_{T_{K}}}^{(K)}$ for the weight of the $S_{T_{K}}$-th SNP for the $T_{K}$-th gene in the K-th tissue). We have also revised the figure legend to explicitly state that SNP prediction weights are derived independently for each tissue. The revisions are highlighted in the Results section (**lines 505, 512-514 on page 25**).

In Tables 1 and 2, adding a “Total” row summarizing the overall number of significant gene-level and gene-tissue pair-level associations across the six binary disease traits would help readers interpret the aggregate findings more easily. This would also make clearer the meaning of the 1,427 (1,355 unique) GWAS risk genes and 479 (470 unique) TWAS risk genes described in the main text.

Thank you for this suggestion. We have added a “Total” row in Table 1 and Table 2 to summarize the overall number of significant gene-level and gene-tissue pair-level associations across the six binary disease traits. The revisions are highlighted in the Results section (**lines 684, 724 on pages 34, 36, respectively**).

In the paragraph describing the third example, “The third example is CCR6 …”, the gene name should be corrected from GCR6 to CCR6 and italicized.

Thank you for your careful review. Upon reviewing our source files, we confirmed that the text is spelled “CCR6”, suggesting the error may have been a visual artifact in the PDF conversion. However, we acknowledge that the gene name was not italicized. We have corrected the formatting to *CCR6* in the revised version (**line 796 on page 39**) and will pay special attention to the converted PDF during the submission process.

**References**

Yang, Jian, et al. "Common SNPs explain a large proportion of the heritability for human height." Nature genetics 42.7 (2010): 565-569.

Zhao, Siming, et al. "Adjusting for genetic confounders in transcriptome-wide association studies improves discovery of risk genes of complex traits." Nature Genetics 56.2 (2024): 336-347.

Parrish, Randy L., et al. "SR-TWAS: leveraging multiple reference panels to improve transcriptome-wide association study power by ensemble machine learning." Nature Communications 15.1 (2024): 6646.
