# Supplementary material for: mFABIO: An integrative multi-tissue TWAS fine-mapping approach to prioritize potentially causal genes and tissues underlying binary traits
Source: PLoS Genet. 2026 May 27;22(5):e1012157. doi: 10.1371/journal.pgen.1012157 (PMC13225670; doi:10.1371/journal.pgen.1012157)
Supplement: Attachment — Submitted filename: response_letter_auresp_2.docx [file pgen.1012157.s014.docx]

**Comments From Reviewer #1:**

In this revision, the authors have been very responsive to the issues raised by the reviewers. I have only minor comments:

1. Page 1, lines 115, it says “These GreX values are assumed to have been pre-computed using standard software such as SuSiE”. Can you double check this is true? My understanding is SuSiE is SNP-based fine mapping method, not for building prediction models for gene expression.

Thank you for your thoughtful comment. Your understanding of SuSiE is correct: SuSiE is primarily a SNP-level fine-mapping method, and genetically regulated expression (GReX) values are typically obtained using established transcriptome prediction frameworks such as elastic net-based models (e.g., PrediXcan). However, because the underlying model used in fine-mapping is a sparse regression model, which is also commonly used for prediction, SuSiE has been recently applied to construct gene expression prediction models through its posterior estimates of cis-eQTL effect sizes. In this manuscript, we followed the existing multi-tissue TWAS fine-mapping method TGFM (Strober et al., 2025) in using SuSiE for generating gene expression prediction models. That said, we agree that referring to SuSiE as “standard software” for generating GReX may be confusing. Therefore, we have revised the manuscript accordingly. The sentence now reads as: “These GReX values are assumed to have been pre-computed using standard transcriptome prediction frameworks such as PrediXcan.” (page 7, lines 114-116).

2. When analyzing multiple tissues, gene expression levels (or their genetically predicted values) across tissues may be highly correlated or even identical, leading to a singular covariance matrix. How do you address this issue in such extreme cases?

Thank you for raising this point. We agree that predicted gene expression (GReX) values across tissues can be highly correlated, and in extreme cases may become nearly identical, which could in principle lead to numerical instability in methods that rely on explicit inversion of covariance matrices. However, our framework is specifically designed to remain well-behaved under such collinearity for several reasons:

First, our method takes advantage of individual-level inputs, and operates directly on the design matrices (i.e., $\hat{\boldsymbol{G}}$ and $\boldsymbol{X}$) within a regression framework and does not require explicit estimation or inversion of a gene-gene covariance matrix. Variational updates are formulated in terms of inner products (e.g., ${\hat{\boldsymbol{G}}}^{\top}\hat{\boldsymbol{G}}$), and regularization is introduced through prior specifications rather than matrix inversion, which avoids numerical issues associated with singular covariance matrices.

Second, as described in the model, all predicted gene-tissue features are standardized to zero mean and unit variance prior to model fitting. While this does not remove collinearity, it ensures consistent scaling and improves numerical stability during optimization.

Finally, we impose a Gaussian ridge prior on SNP effects and a structured sparse prior (SuSiE) on gene-tissue effects. These priors act as regularization, preventing overfitting and stabilizing inference even when predictors are highly correlated. Critically, the SuSiE prior is designed to operate under extreme collinearity. Each single-effect component selects one feature, forcing highly correlated gene-tissue predictors to compete within the same component. When multiple features are nearly identical, the model distributes posterior inclusion probability (PIP) across them rather than attempting to distinguish indistinguishable effects. This yields stable and interpretable results even when the design matrix is rank-deficient.

We have clarified this point in the revised manuscript to emphasize that our method is robust to highly correlated or even identical predicted expression features and does not require invertible covariance matrices (pages 10-11, lines 187-199).

3. In a single LD region, the number of SNPs analyzed can exceed 1,000. Does this pose any computational challenges?

Thank you for this question. We agree that in a single LD region, the number of SNPs can exceed 1,000. However, our framework is designed to remain computationally efficient in this setting.

First, the direct SNP effects are modeled using a Gaussian prior, which treats SNP effects jointly rather than performing discrete variable selection over all SNPs. This avoids the computational complexity that would arise from selecting among a large number of SNPs and allows updates to be computed efficiently using matrix operations.

Second, our inference is based on coordinate ascent variational inference (CAVI), where updates for SNP effects depend primarily on inner products involving the genotype matrix (e.g., $\boldsymbol{X}^{\top}\boldsymbol{X}$). These quantities can be computed efficiently and reused across iterations, leading to scalable performance. Importantly, the overall computational complexity scales linearly with the number of SNPs (i.e., $O(N\times P)$) for the SNP component.

Third, the sparse modeling and fine-mapping step is performed at the level of gene-tissue features via the SuSiE prior, rather than at the SNP level. As a result, the computing burden is confined to a much smaller feature space (number of gene-tissue pairs), while the SNP-level component serves as a regularized background adjustment.

In practice, we find that analyzing regions with thousands of SNPs does not pose a significant computational bottleneck. We have previously included a section of “Benchmark of runtime and memory usage” in the Results (page 41, lines 818-836), which shows that our method is scalable to biobank-scale datasets, and has competitive computational efficiency compared with other methods. In response to this comment, we have added the average number of SNPs per LD region to that section (page 41, line 830) to provide additional context.

4. In model (1) or (3), the authors assume both the intercept mu and the residue follow normal distributions. How do they use difference information from the data?

Thank you for this question. We would like to clarify that while the intercept ($\mu$) and the residual term ($\epsilon_{i}$) are assigned Gaussian distributions as part of the probit (liability threshold) formulation, the model fully leverages the information in the observed binary outcomes through the likelihood.

Specifically, under the probit model in Equation (1), the probability of observing the binary outcome $y_{i}$ is linked to the linear predictor through the cumulative distribution function of the standard normal distribution $N(0,1)$. In the equivalent latent liability representation (Equations (2)–(3)), the observed binary outcome imposes a truncation constraint on the latent variable $z_{i}$:

- if $y_{i}=1$, then $z_{i}>0$
- if $y_{i}=0$, then $z_{i}\leq0$

As a result, the posterior distribution of $z_{i}$ becomes a truncated normal distribution, and its expectation depends explicitly on the observed outcome $y_{i}$. This is the primary mechanism through which the model incorporates the difference between cases and controls.

The intercept $\mu$ in the model plays a key role in this process as a global baseline liability parameter. It shifts the mean of the latent variable $z_{i}$ and thus controls the baseline probability of $y_{i}=1$. Importantly, $\mu$ is not determined by its prior; rather, it is estimated from the data through the likelihood. Because the observed outcomes constrain $z_{i}$ via truncation, the model adjusts $\mu$ so that the implied distribution of $z_{i}$ matches the observed case-control proportion. For example, in settings with rare diseases (many $y_{i}=0$), $\mu$ will be inferred to be negative, shifting most liabilities below zero, and vice versa for more prevalent traits.

During variational inference, the posterior of $\mu$ is updated using the expected latent liabilities $E[z_{i}]$, which themselves depend on the observed outcomes through truncation. Therefore, both $\mu$ and $z_{i}$ are directly informed by the data, ensuring that the model meaningfully captures differences between individuals with different phenotypes.

In summary, although Gaussian assumptions are used for the residuals and priors for computational tractability and identifiability, the likelihood induced by the observed binary outcomes, together with the truncation of the latent liabilities, ensures that the model fully utilizes the information in the data.

Page 10, line 181: Assuming that the vector γ follows a multinomial distribution, Multinomial(1,π), implies that there is at least one causal gene in the region under consideration. If mFABIO is applied to all regions without pre-screening (e.g., using marginal TWAS to identify candidate regions likely to contain causal genes), it may identify putative causal genes in regions that do not truly harbor any causal genes. Please clarify how mFABIO avoids such false positives.

Thank you for this comment. You are correct that, under the SuSiE prior, each single-effect component $\boldsymbol{\gamma}_{l}\sim\text{Multinomial}(1,\boldsymbol{\pi})$ selects exactly one feature *a priori*. However, this does not imply that the model forces the presence of a causal gene in every region. Specifically, while each component selects a gene-tissue pair, the corresponding effect size $\beta_{l}$ is modeled with a zero-centered Gaussian prior:

$$\beta_{l}\sim N(0,\sigma_{\beta}^{2}).$$

As a result, the model allows $\beta_{l}$ to shrink toward zero when there is no supporting signal in the data. In regions with no true causal gene, the posterior distribution will concentrate around $\beta_{l}\approx0$ for all components, effectively representing a null signal despite the multinomial assignment. Below is an example boxplot to show that, combining the 100 replicates of our baseline null simulation, the posterior mean of each component-specific effect $\beta_{l}$ term across *L* (*=* 10 by default) components are indeed concentrated around 0:

Furthermore, the posterior inclusion probabilities (PIPs) are driven by the data through the variational updates. In the absence of a true association signal, PIPs tend to be diffuse and low across all gene-tissue pairs, rather than concentrating on a specific feature. Therefore, although the model structurally assigns each component to a feature, it will not produce any strong signals when the data do not support them, and the inference is usually based on the magnitude of PIPs and credible sets rather than the mere existence of a selected component. Below is an example histogram to show that, combining the 100 replicates of our baseline null simulation, the feature-level PIPs are indeed low with a majority of 0:

We also agree with your point that incorporating a pre-screening step is important. In fact, using marginal TWAS to identify candidate regions likely to contain causal genes is a standard practice prior to gene-level fine-mapping. In this manuscript, we also followed the TGFM strategy of restricting the fine-mapping analysis to gene-tissue pairs with reliable genetic prediction by using the “purity filter” from SuSiE during the eQTL modeling step. Specifically, this filter removes gene-tissue pairs for which all components defining the prediction model contain variants with a minimum absolute correlation below 0.5 within the 95% credible set, thereby ensuring well-conditioned and biologically meaningful predictors.

Finally, we note that we have previously included a section of “mFABIO controls false signals in null simulations” in the Results (pages 26-27, lines 538-551), which demonstrate that mFABIO effectively controls false positives across multiple null simulation settings, in which regions do not contain any true causal genes.

**References**

Strober, Benjamin J., et al. "Fine-mapping causal tissues and genes at disease-associated loci." *Nature genetics* 57.1 (2025): 42-52.
